# When Feelings Arise with Meanings: How Emotion and Meaning of a Native Language Affect Second Language Processing in Adult Learners

**DOI:** 10.1371/journal.pone.0144576

**Published:** 2015-12-10

**Authors:** Agnes Sianipar, Renée Middelburg, Ton Dijkstra

**Affiliations:** 1 Radboud University Nijmegen, Donders Institute of Brain, Cognition and Behaviour, Centre for Cognition, Montessorilaan 3, 6525 HR, Nijmegen, The Netherlands; 2 Faculty of Psychology, Universitas Indonesia, Depok, Jawa Barat, Indonesia; Leiden University, NETHERLANDS

## Abstract

To determine when and how L2 learners start to process L2 words affectively and semantically, we conducted a longitudinal study on their interaction in adult L2 learners. In four test sessions, spanning half a year of L2 learning, we monitored behavioral and ERP learning-related changes for one and the same set of words by means of a primed lexical-decision paradigm with L1 primes and L2 targets. Sensitivity rates, accuracy rates, RTs, and N400 amplitude to L2 words and pseudowords improved significantly across sessions. A semantic priming effect (e.g, prime “driver”facilitating response to target “street”) was found in accuracy rates and RTs when collapsing Sessions 1 to 4, while this effect modulated ERP amplitudes within the first 300 ms of L2 target processing. An overall affective priming effect (e.g., “sweet” facilitating”taste”) was also found in RTs and ERPs (posterior P1). Importantly, the ERPs showed an L2 valence effect across sessions (e.g., positive words were easier to process than neutral words), indicating that L2 learners were sensitive to L2 affective meaning. Semantic and affective priming interacted in the N400 time-window only in Session 4, implying that they affected meaning integration during L2 immersion together. The results suggest that L1 and L2 are initially processed semantically and affectively via relatively separate channels that are more and more linked contingent on L2 exposure.

## Introduction

The importance of foreign language expertise to diverse strands of life (social, cultural, and professional, to name a few) motivates many adults to learn a new language at a later age. The ability to communicate efficiently and appropriately as an L2 speaker requires vocabulary knowledge that contains both the conceptual/semantic and connotative/affective meaning of words used by conversation partners [1]. However, up till now, most longitudinal studies on L2 acquisition in adults have only focused on the development of mappings between L2 word forms and their semantic meanings. These studies have shown that foreign word learning is accompanied by rapid changes in adults’ representational structures and brain responses (EEG), as well as in accuracy and reaction time (RT). Nevertheless, how and when an adult L2 learner additionally acquires the emotional properties of a word’s meaning during L2 learning and immersion remains an open question. This topic, linking emotion to cognition in L2 learners, is investigated in the present study.

### Affective Priming in monolinguals and bilinguals

Considering words like ‘happy’, ‘shark’, and ‘book’ makes clear that lexical representations are not only associated with a semantic meaning (concept) but also have an affective value (e.g., valence or arousal). De Houwer and Hermans [2] conceptualized the affective value of a word as an affective tag (e.g., positive, negative) linked to a concept/semantic node in the semantic network. Thus, the semantic meaning of the word ‘sun’ (i.e., the representation of the object *sun*) is linked to its affective meaning (it has positive valence) and the two representations constitute different yet integrated aspects of word meaning.

The affective networks between word concepts have been studied by means of the so-called ‘affective priming’ technique [3]. In sequential affective priming, the affective congruence between a prime and a target is manipulated. An affective priming effect is obtained when affective responses to targets (e.g., deciding whether a target is positive or negative) following affectively congruent primes have shorter latencies and higher accuracies than following affectively incongruent primes. The mechanism underlying affective priming is still under debate [4]. Some researchers argue that affective priming effects come about via a spreading activation mechanism that is analogous to the underlying mechanism of sequential semantic priming for studying semantic memory organization [5]. The activation of a positively-tagged representation should spread to other positively-tagged representations, but might inhibit negatively-tagged representations [2]. Affective priming has been used to investigate the relationship between cognition and emotion at a representational level [6]. Effects of affective congruency have generally been considered as an indication that affective representations (e.g., valence or arousal) are processed automatically.

However, RT studies that systematically manipulated both semantic and affective congruence within a language (a comparative approach) have led to conflicting results. Storbeck and Robinson [7] found semantic priming effects in lexical decision and evaluative decision tasks that were not modulated by affective congruence. In contrast, Ferré and Sanchez-Casas [8] manipulated the semantic similarity between primes and targets in lexical decision, orthogonally to affective congruence, and observed that semantic relatedness was modulated by affective congruence and target valence, while both priming effects were also significant on their own. We note that the stimuli in Storbeck and Robinson’s study were not drawn from available affective norms of English words, which might explain the deviating results between the two studies.

Castner et al. [9] manipulated semantic associativeness, instead of semantic similarity, and affective congruence of primes and targets. In their study, semantic and affective priming interactively modulated the RTs of their control participants. Affective priming was larger for unrelated prime-target combinations and semantic priming was larger for incongruent prime-target combinations. Note that Castner et al. employed negative and neutral items. The findings from Castner et al. [9] and Ferré and Sanchez-Casas [8] suggest that in a native language semantic and affective representations of words are linked and may interact. These findings are in line with previous studies showing that the affective congruence of primes and targets modulates targets’ semantic categorization process [10] or that the semantic relatedness of primes and targets from different affective categories interfered with the lexical decision on targets [11]. They raise the question to what extent lexical decisions of semantically related or unrelated prime-target pairs are interfered with by their affective congruence. For example, affective priming might be reduced when there is a competition between the prime’s and target’s semantic relatedness and affective congruence; in contrast, affective priming might be increased when the primes and targets are semantically related and affectively congruent [4]. Hence, even though semantic associativeness and affective congruence between primes and targets refer to different aspects of the conceptual meaning system, they are also coupled in such a way that an exchange of conflicting information may result in a reduction of the semantic priming effect in a mismatch situation.

These studies and many others were carried out to understand the relationship between affective and semantic meaning representations in L1. Their findings raise the question whether an interaction of semantic and affective representational networks can also occur across languages. Specifically, while in native language acquisition semantics and affect appear to pass through a parallel development that is strongly linked, this relationship might be weaker or even absent in late bilinguals and might be sensitive to L2 proficiency and L2 exposure [12].

Altarriba and Canary [13] investigated the relationship between cognition and affective processes in L1 and L2 semantic networks in terms of arousal effects on word processing. In a within-language semantic priming study with L1 and fluent L2 speakers, they tested to what extent a high-arousing target’s lexical decision was slowed by the arousal congruency and relatedness of a preceding prime. Facilitation arose for L1 and L2 targets preceded by highly and moderately arousing primes, but not for targets preceded by non-arousing primes. L1 and L2 differences in arousal effects on target words were also obtained. However, although this shows that semantic networks in L1 and L2 are moderated by arousal congruence between primes and targets, Altarriba and Canary’s design did not allow them to dissociate the effect of arousal and semantic relatedness, because their unrelated primes were always non-arousing words. Hence, the interconnectedness of arousing and non-arousing words was not fully resolved through this design.

### The present study: Linking L2 affective processes to L2 word learning

An L2 is often learned at a later age in relatively neutral class-room situations. This is quite different from the early learning of an L1, where emotions are part and parcel of the communicative situation. Nevertheless, several studies indicate that emotional valence has a role to play in bilingual processing and in L2 learning. For instance, a study by Harris, Ayçiçegi, and Gleason [14] showed that different types of emotional stimuli modulated L2-dominant speakers’ skin conductance responses (SCR) to both L1 and L2 verbal stimuli. The emotion effect was stronger in L1 when the stimuli were negative and presented as childhood reprimands. However, in general, emotion effects were also present in SCR to L2 stimuli. These findings suggest that both age of acquisition and L2 proficiency/exposure affect the extent to which emotion influences L2 speakers’ sensitivity to both auditory and visual word stimuli.

Many subsequent studies have investigated the extent to which emotionality is reduced in L2. However, most of these studies did not control at the same time for a possible confounding of the effects of age of acquisition, and levels of L2 exposure / L2 proficiency. For example, Degner, Doycheva and Wentura [15] reported a valence priming facilitation effect in highly-proficient L2 speakers with high levels of immersion and frequency of L2 use. This finding suggests that the emotional associations of L2 representations might indeed be anchored in the bilinguals’ experiences with L2. However, the participants of this study varied in terms of their age of L2 acquisition, with some people already starting learning their L2 before the age of 8. Furthermore, none of the available studies clarified whether the effect of word valence already arises during the very early stages of learning. Hence, we conducted a longitudinal class-room study involving a coherent group of adult L2 learners set out to clarify the time-course and interaction of the developing meaning and emotional valence effects in a new language.

Our cross-language priming study had three aims: (1) To determine behavioral and neural correlates of the interconnectedness of emotion-laden words from different languages, thus extending the previous studies on the relationship between cognition and affective processes in L1 and L2 semantic networks [13,15]; (2) To assess when valence effects of L2 words arise during L2 learning and L2 processing; (3) To pinpoint the interaction of semantic and affective meaning in L2 learning and processing by means of ERPs.

We applied the comparative approach by Storbeck and Robinson [7] to dissociate the influence of semantic and affective properties of L1 words on L2 semantic and affective processing. This approach can also clarify whether affective properties of L2 words have already modulated neural and behavioral responses during an early stage of L2 learning in a longitudinal learning paradigm as used by McLaughlin, Osterhout, and Kim [16]. When L1 words serve as primes, they may not only activate semantic but also affective meanings of L1 targets, suggesting an integration of semantic and affective word meanings. For L2 targets, such a semantic integration process might be absent when semantic and affective meanings are not linked yet, or when the affective meanings are not accessible yet. Both situations might occur during the very early stages of L2 learning, when L2 learners have just encountered and learned L2 word forms and their semantic meaning. However, in an immersion setting, L2 words would be more frequently used, allowing for the establishment of richer knowledge representations, including emotional valence, to be linked to the word forms and the semantic meaning. This issue is the specific case being investigated in the current study.

Our longitudinal study examined the development of L2 semantic and affective meaning during the very early phases of learning over a period of 6 months. We combined semantic and affective priming paradigms and employed the lexical decision task (LDT), used in most priming studies. The LDT was helpful in avoiding any task-relevant influence on conceptual or affective responses towards targets, given that the task does not emphasize the relatedness of the conceptual or affective meanings of primes and targets [7,9,17]. We recorded event-related potentials to allow relatively direct, on-line measures of the learning rates of L2 words [16]. In addition, we analyzed RTs and accuracy rates, as well as sensitivity rates (e.g., *d*-prime scores), to test the sensitivity of our paradigm in detecting changes in learner’ sensitivity across sessions.

In the ERP analysis of each session, we considered ERP components associated with different stages of word processing [18] that are also sensitive to semantic and/or affective meanings of words. Early components we considered were N1/P1 (50–100 ms), related to the early perceptual process [19–22] and P2/N2 (150–300 ms), related to the process of attentional orienting to semantic features and meaning accessibility in learning [23,24]. Late-latency components were the N400 (300–600 ms), related to semantic/contextual integration [18,25] and the LPC (500–800 ms), associated with emotional evaluative process or sustained attention to emotion conflict [26,27].

We made the following predictions with respect to the learning aspect of L2 processing. In our study, L2 positive and neutral words were employed as targets. Previous findings in L1 studies suggest that positive words are responded to faster [28] and more accurately [29,30] than neutral and negative words. In bilinguals, positive words are also better encoded in memory compared to both negative and neutral words irrespective of language dominance [31]. We therefore predicted that, relative to neutral L2 words, positive L2 words would yield faster RTs and higher accuracy rates and that this effect would emerge towards the end of the language course (third session). Different neural correlates should arise for L2 neutral and positive targets in the course of L2 learning and would also be reflected in amplitude differences in early and late ERP components from the third session onwards. Intuitively, one would expect that L2 forms learned in classroom settings with low-level frequency of usage would not initially be able to activate semantic/conceptual and valence associations. However, we predicted a valence effect to arise in the third session (at the end of the language course) and later based on the assumption that emotional associations emerge in the context where L2 stimuli are learned [32] and also depend on the frequency of usage of L2 words [15]. If a valence effect would nevertheless already arise in the first session, it would be an indication that *from the earliest stage onwards*, L2 forms can activate semantic concepts and their valence associations, which were previously linked only with L1 forms. Evidence for this prediction would underline the importance of valence as a defining aspect of a conceptual representation that is linked to both languages of a bilingual.

Furthermore, in light of the evidence of the interacting semantic and affective priming in L1 studies and the view of interrelated semantic conceptual development and affective linguistic conditioning in language learning [12], we expected an interaction of semantic and affective priming in late ERP components that increases across sessions with additional L2 exposure. According to the latter hypothesis, semantic and affective representations of L2 words become more strongly linked when the learners are immersed in an L2 environment, allowing emotional and contextualized processes to exhibit increasingly stronger affective interference effects on semantic processing.

## Materials and Method

### Participants

We invited 32 right-handed German native speakers who came to follow a 5-week Dutch course at Radboud University Nijmegen (mean age: 19.6, range: 18–25, 8 males/24 females) in preparation of a Dutch competency examination as part of the entry requirements for university study. Before being accepted as a participant, each person was screened to make sure he/she did not have any experience with Dutch before coming to study in the Netherlands. The online measurements (RTs and ERP recordings) were conducted in 4 sessions. Session 1 took place between 5–12 days after arrival (one week upon following the course), Session 2 between 26–33 days after arrival (the last week of the course), Session 3 between 79–90 days after arrival (immersion period), and Session 4 between 156–171 days after arrival (immersion period). The first session involved 32 participants, the second 31 participants, the third 29 participants, and the fourth 24 participants. Only measurements from 24 participants (mean age: 19.5, range 18–25, 6 males) who attended all 4 sessions were considered in the analyses. These 24 participants all passed the test for the *Staatexamen NT2* (State exam for Dutch as a foreign language) and were accepted as students at the Radboud University Nijmegen at the end of Session 3. All participants received either monetary or credit compensation.

### Ethics Statement

This study was approved by the Ethics Committee of the Faculty of Social Sciences (ECSW) of Radboud University Nijmegen (ECG2012-3008-043: Verwerving van een vreemde taal) on August 30th, 2012. All participants provided their written informed consent to participate in this study.

### Stimulus Materials and Design

Stimulus materials consisted of 320 prime-target pairs, with German words in the participant’s native language (German) as primes. Targets were 40 words in the newly learned language (Dutch) for which the prime-target relations were manipulated with respect to semantic relatedness and emotional congruency (resulting in 2 x 2 x 40 = 160 word targets). The D-ANEW corpus was used as database source to derive ratings on the valence, arousal, and imageability levels of Dutch words, following Jasmin and Casasanto [33]. D-ANEW is a corpus of 1,031 Dutch translation equivalents of the Affective Norms for English Words [34]. We selected the Dutch target stimuli by screening all Dutch vocabulary that the participants were obliged to learn from the Contact! textbook in the first two weeks of their language course. Next, we selected our target words based on those learned words that were also included in D-ANEW corpus. As mentioned before, we selected both positive and neutral Dutch targets. In addition, we created additional 160 orthographically and phonologically legal Dutch non-words from a different list of Dutch words by changing one or two their letters. The Dutch target words and non-words were presented with all capital letters. The selected positive and neutral target words differed in their lengths, valence and arousal levels (all *p*s ≤ .001). However, the two groups did not show any significant difference in imageability and SUBTLEX-NL [35] frequency levels (all *p*s > .1).

All German primes in this study were derived from Berlin Affective Word List–Reloaded/BAWL-R [36]. Throughout the experiment, German nouns were written with a capital letter followed by lower case letters while German adjectives and verbs were written with lower case letters. The German stimuli were divided into three different groups of primes based on their emotional valence. In total, there were 160 negative primes, 80 neutral primes, and 80 positive primes. Negative, positive, and neutral primes differed significantly in their valence and arousal levels (all *p*s < .001). The arousal levels between positive and neutral primes were not significant (*p* > .1). Furthermore, the primes were matched in their imageability and SUBTLEX-DE [37] frequency levels (all *p*s > .1). The stimuli with their characteristics are presented in S1 Table.

Each word target appeared 4 times in every session and for each trial, its appearance being preceded by one of the three valence variants of L2 primes, which were assigned based on the four types of prime-target relationships. Primes and targets were (1) semantically associated and affectively congruent; (2) semantically associated, but affectively incongruent; (3) semantically unassociated, but affectively congruent; and (4) semantically unassociated and affectively incongruent (control) (Table 1). Each pseudoword target was also preceded by an L1 prime from one of three prime valence categories.

**Table 1:** The experiment design and stimulus sample.

Affective Congruence	Semantic Relatedness	Prime Valence	Target Valence	Example stimuli	
				L1 Prime	L2 Target
Congruent	Related	Positive	Positive	süß	SMAAK
Congruent	Related	Neutral	Neutral	Fahrer	STRAAT
Congruent	Unrelated	Positive	Positive	Lohn	SMAAK
Congruent	Unrelated	Neutral	Neutral	Neugier	STRAAT
Incongruent	Related	Negative	Positive	sauer	SMAAK
Incongruent	Related	Negative	Neutral	Stau	STRAAT
Incongruent	Unrelated	Negative	Positive	Natter	SMAAK
Incongruent	Unrelated	Negative	Neutral	Bahre	STRAAT

An additional rating study was run with 22 German-Dutch bilingual speakers from Radboud University Nijmegen who never participated in the main study. Participants were asked to rate the associativeness between primes and targets on a 1-to-7 Likert Scale with 7 being the index for the “most associated pairs”. Participants rated semantically associated prime-target pairs as significantly more associated (mean associative = 5.6, SD = .7) than semantically unassociated pairs (mean unassociated = 1.4, SD = .6, *t* (159) = -28.94, *p* < .001). Semantically associated and emotionally congruent prime-target pairs did not differ significantly in their associativeness ratings (mean associated and congruent = 5.7, SD = .8) from semantically associated but incongruent prime-target pairs (mean associated and incongruent = 5.5, SD = .6, *t* (79) = -1.11, *p* = .14). The associativeness ratings between semantically unassociated and affectively congruent prime-target pairs (mean unassociated and congruent = 1.43, SD = .41) and semantically unassociated and incongruent prime-target pairs (mean unassociated and incongruent = 1.38, SD = .39, *t* (79) = .12, *p* = .54) were also not statistically different.

Prior to data acquisition, all prime-target pairs were first divided into 4 sublists in which each target word appeared only once. Each sublist consisted of 40 different target words and 40 different target non-words. For each participant, each sublist was randomized and then combined with the other three sublists to form an individual stimulus list. Thus, participants were presented with different orders of sub-randomized stimuli in which a target word was repeated in a different prime-target relation condition after at least 80 trials. Every target non-word appeared only once in each stimulus list.

### Procedure

After electrode application, participants were seated in front of a computer screen in a dimly lit booth. The viewing distance was approximately 1 m. Stimuli were presented individually at the centre of the screen in serial visual presentation mode. Trials began with a fixation cross (duration 500 ms) followed by a 300 ms blank screen after which a prime appeared for 250 ms. After the prime disappeared, a blank screen appeared for 350 ms followed by the appearance of a target. Thus, stimulus-onset asynchrony (SOA) was 600 ms. The target word stays on the screen until the participant responded (or at the maximum 3000 ms) and the inter-trial interval lasted 1500 ms. Using a button box, participants were asked to judge whether the target was a Dutch word or not by pressing the left button for “non-word” or the right button for “word”. They were instructed to make eye movements after they responded toward a target or in between trials. The trials were grouped in 3 blocks. Each block lasted about 8 minutes and participants were given short breaks in between.

### EEG Recordings

EEG was recorded from 25 active Ag/AgCl electrodes (Acticap), located according to the extended 10–20 electrode systems. An additional electrode was placed at the right mastoid as well as another electrode at the left mastoid as the reference. Five electrodes were placed over midline sites Fz, FCz, Cz, Pz, and Oz. Ten additional electrode pairs were placed over the lateral sites: FP1/FP2, F3/F4, FC5/FC6, FC1/FC2, T7/T8, C3/C4, CP5/CP6, CP1/CP2, P7/P8, and O1/O2. Vertical eye movements were monitored through two additional electrodes on the infraorbital and supraorbital ridge below and above the left eye, respectively. Horizontal eye movements were monitored through another two electrodes; each was placed approximately at left and right outer canthi. The ground electrode was placed on the forehead. Electrode impedance was kept below 20 kΩ. EEG and EOG recordings were amplified through BrainAmp DC amplifier (Brain Products, Germany), using a 125 Hz low-pass filter, a sampling frequency of 500 Hz, a time constant of 10 Hz, and stored for subsequent off-line analysis using BrainVision Analyzer 2.0 software (Brain Products, Germany).

For offline analysis, EEG data were re-referenced to the average of both mastoids. Signals from the EOG-electrodes were converted into bipolar horizontal and vertical EOG signals. The data were digitally filtered at 0.1 Hz (high-pass) and 30 Hz (low-pass) Butterworth filter. An additional 50-Hz notch filter was applied in order to remove line noise. A linked eye-channel was created off-line by re-referencing the left vertical EOG to the left horizontal EOG. Ocular artifacts were corrected using the Gratton-Coles algorithm [38]. The continuous EEG was then segmented to epochs of 1000-ms from stimuli onset for the primes and 1000-ms from stimuli onset for the targets, with a 200-ms baseline correction. Artifact detection was performed using ± 75μV peak-to-peak mean amplitude criterion and all segments affected by residual artifacts were semi-automatically rejected. Across four sessions, 20 participants were included while four participants were excluded from ERP analysis due to having more than 20% artifact rates (final N = 20). Artifact-free segments were averaged across conditions and participants. The number of trials (only correctly answered trials were included) per condition was on average 18 (SD = 1.9).

For statistical analysis, we conducted repeated measure ANOVAs using six quadrants as followed: Left anterior (F3, FC1, FC5), right anterior (F4, FC2, FC6), left central (C3, CP1, CP5), right central (C4, CP2, CP6), left posterior (P3, P7, O1) and right posterior (P4, P8, O2). Separate ANOVAs would be carried out to assess the effect at the midline (Fz, Cz, Pz). The statistical analysis consisted of three parts. Firstly, we assessed participants’ sensitivity toward the valence levels of prime stimuli in Late Posterior Complex/LPC (430–550 ms post prime on-set) time-window across sessions. This ERP analysis considered the extent to which our participants’ attention was spontaneously directed to the emotional content of the L1 primes during silent reading [39]. Secondly, we investigated N400 effects (350–550 ms) on targets’ lexical status (words vs. nonwords) across sessions. The results of the ERPs of the primes’ and targets’ lexicality are provided in S2 Text attached to this paper.

Thirdly, we examined the extent to which the ERPs for L2 word target processing were influenced by affective congruence and semantic relatedness between the L1 primes and the L2 targets in several time-windows (based on visual inspections): 80–150 ms (N1/P1), 150–300 ms (P2/N2), 350–550 ms (N400), and 550–700 ms (LPC). For each time-window, a repeated measure ANOVA involving 4 (session) x 2 (affective congruence) x 2 (semantic relatedness) x 2 (target valence) x 6 (quadrant) as within-subject factors was conducted to explore effects on L2 target processing over the quadrants, while a separate repeated measure ANOVA, which involves 4 (session) x 2 (affective congruence) x 2 (semantic relatedness) x 2 (target valence) as within-subject factors were additionally conducted for midline analysis for each time-window. In all ANOVAs, Huynh-Feldt correction was applied to the degrees of freedom in order to correct for violations of the sphericity assumption. Uncorrected degrees of freedom and corrected *p*-values are reported.

## Results

Before we analyzed the ERP data of the four sessions, we performed a number of preliminary analyses to check the sensitivity rate, accuracy, and RTs of the participants in each session. Repeated measure ANOVAs were conducted for these different behavioral dependent variables. Given space restrictions, they are reported in S1 Text associated with this paper. We will present a short overview of these results here before zooming in on our ERP analyses.

### L1-L2 priming effects on Accuracy Rates

A 4 (session) x 2 (semantic relatedness) x 2 (affective congruence) x 2 (target valence) repeated measures ANOVA over accuracy rates from 24 subjects revealed a significant main effect of semantic relatedness (*F* (1, 23) = 4.51, *p* = .045, η_p_^2^ = .16), indicating that more errors occurred in judging the lexicality of L2 targets following L1 unrelated primes (mean accuracy = 94.17%, SD = 2.83) than following L1 related primes (mean accuracy = 94.74%, SD = 2.48). There were no significant main effect of affective congruency or other significant interaction effects on accuracy rates (all *p*s > .1).

### L1-L2 priming effects on Reaction Times

A repeated measures ANOVA with the same factors as before revealed a significant main effect of semantic relatedness (*F* (1, 23) = 5.69, *p* = .03, η_p_^2^ = .2), showing somewhat shorter RTs for L2 targets following L1 related primes (mean = 588 ms, SD = 60 ms) than following L1 unrelated primes (mean = 593 ms, SD = 62 ms). The main effect of affective congruence (*F* (1, 23) = 6.76, *p* = .02, η_p_^2^ = .23) was also significant, reflecting that RTs for L2 targets following L1 congruent primes (mean = 588 ms, SD = 62 ms) were significantly shorter than following L1 incongruent primes (mean = 592 ms, SD = 60 ms). However, the interaction effects of semantic relatedness by affective congruence and of semantic relatedness or/and affective congruence by session were not significant (all *p*s > .1).

### Event-Related-Potentials / ERPs

The F-values and effect size of our ERP findings are presented in Table 2 (N1/P1 and P2/N2) and Table 3 (N400 and LPC). The means and standard deviations for the observed effects are presented in Table 4 (N1/P1 time-window), Table 5 (P2/N2 time-window), Table 6 (N400 time-window), and Table 7 (LPC time-window).

**Table 2:** The F-values and effect size in N1/P1 and P2/N2 time-windows.

*SOURCES*	*df*	80–150 ms (N1/P1)				150–300 ms (P2/N2)			
		*Quadrant*		*Midline*		*Quadrant*		*Midline*	
		*F*	*np2*	*F*	*np2*	*F*	*np2*	*F*	*np2*
**Quadrant x Affective Congruence**	*3*,*57*	6.64**	0.26	< 2.50	< 0.12	< 2.50	< 0.12	< 2.50	< 0.12
Left Anterior	*1*,*19*	< 2.50	< 0.12						
Right Anterior	*1*,*19*	< 2.50	< 0.12						
Left Central	*1*,*19*	< 2.50	< 0.12						
Right Central	*1*,*19*	< 2.50	< 0.12						
Left Posterior	*1*,*19*	4.30#	0.18						
Right Posterior	*1*,*19*	5.10*	0.20						
**Semantic Relatedness**	*1*,*19*	25.64***	0.57	21.29***	0.54	12.51**	0.40	11.41**	0.37
**Session x Semantic Relatedness**	*3*,*57*	< 2.50	< 0.12	< 2.50	< 0.12	2.91*	0.13	3.78*	0.17
Session 1	*1*,*19*					< 2.50	< 0.12	< 2.50	< 0.12
Session 2	*1*,*19*					13.97**	0.42	12.82**	0.40
Session 3	*1*,*19*					5.28*	0.22	< 2.50	< 0.12
Session 4	*1*,*19*					< 2.50	< 0.12	4.60*	0.20
**Quadrant x Semantic Relatedness**	*5*,*95*	7.85***	0.29	< 2.50	< 0.12	3.25*	0.15	< 2.50	< 0.12
Left Anterior	*1*,*19*	20.64***	0.52			6.76*	0.26		
Right Anterior	*1*,*19*	25.90***	0.58			13.11**	0.40		
Left Central	*1*,*19*	15.16**	0.44			6.40*	0.25		
Right Central	*1*,*19*	30.13***	0.61			12.90**	0.40		
Left Posterior	*1*,*19*	12.68**	0.40			7.70*	0.29		
Right Posterior	*1*,*19*	30.30***	0.61			22.10***	0.58		
**Target Valence**	*1*,*19*	10.60**	0.36	16.05**	0.46	< 2.50	< 0.12	< 2.50	< 0.12
**Quadrant x Target Valence**	*5*,*95*	4.44**	0.19	< 2.50	< 0.12	< 2.50	< 0.12	< 2.50	< 0.12
Left Anterior	*1*,*19*	10.60**	0.36						
Right Anterior	*1*,*19*	15.80**	0.46						
Left Central	*1*,*19*	9.20**	0.33						
Right Central	*1*,*19*	13.28**	0.41						
Left Posterior	*1*,*19*	< 2.50	< 0.12						
Right Posterior	*1*,*19*	4.74*	0.2						

Note

***p < .001

**p < .01

*p < .05

#*p* < .1

**Table 3:** The F-values and effect size in N400 and LPC time-windows.

*SOURCES*	*df*	350–550 ms (N400)				550–700 ms (LPC)			
		*Quadrant*		*Midline*		*Quadrant*		*Midline*	
		*F*	*np2*	*F*	*np2*	*F*	*np2*	*F*	*np2*
**Session**	*3*,*57*	8.10***	0.30	6.52***	0.26	5.70***	0.23	4.14*	0.18
Session 1 vs. Session 2	*1*,*19*	12.41**	0.40	11.23**	0.37	< 2.50	< 0.12	< 2.50	< 0.12
Session 2 vs. Session 3	*1*,*19*	< 2.50	< 0.12	< 2.50	< 0.12	13.98**	0.42	12.82**	0.40
Session 3 vs. Session 4	*1*,*19*	< 2.50	< 0.12	< 2.50	< 0.12	5.28*	0.22	< 2.50	< 0.12
**Target Valence**	*1*,*19*	27.63***	0.59	34.82***	0.65	6.88*	0.27	6.42*	0.25
**Session x Affective Congruence x Semantic Relatedness**	*3*,*57*	3.13*	0.14	2.64#	0.15	4.99**	0.21	3.96*	0.17
Session 1: Congruence x Relatedness	*1*,*19*	< 2.50	< 0.12			< 2.50	< 0.12	< 2.50	< 0.12
Session 2: Congruence x Relatedness	*1*,*19*	< 2.50	< 0.12			< 2.50	< 0.12	< 2.50	< 0.12
Session 3: Congruence x Relatedness	*1*,*19*	< 2.50	< 0.12			3.70#	0.16	4.15#	0.18
Session 4: Congruence x Relatedness	*1*,*19*	18.18***	0.49			8.67**	0.31	6.12*	0.24
Related: Incongruent vs. Congruent	*1*,*19*	12.45**	0.40			< 2.50	< 0.12	< 2.50	< 0.12
Unrelated: Incongruent vs. Congruent	*1*,*19*	6.26*	0.25			5.80*	0.23	5.52*	0.23
Congruent: Unrelated vs. Related	*1*,*19*	11.37**	0.37			< 2.50	< 0.12	< 2.50	< 0.12
Incongruent: Unrelated vs. Related	*1*,*19*	< 2.50	< 0.12			9.65**	0.34	5.58*	0.23
**Affective Congruence x Target Valence**	*1*,*19*	4.57*	0.19	< 2.50	< 0.12	< 2.50	< 0.12	< 2.50	< 0.12
Congruent: Neutral vs. Positive	*1*,*19*	11.05**	0.37						
Incongruent: Neutral vs. Positive	*1*,*19*	33.89***	0.64						
**Session x Semantic Relatedness x Target Valence**	*3*,*57*	3.13*	0.14	< 2.50	< 0.12	3.25*	0.15	2.79*	0.13
Session 1: Semantic Relatedness x Target Valence	*1*,*19*	< 2.50	< 0.12			< 2.50	< 0.12	< 2.50	< 0.12
Target Valence	*1*,*19*	6.57*	0.26			< 2.50	< 0.12	< 2.50	< 0.12
Session 2: Semantic Relatedness x Target Valence	*1*,*19*	< 2.50	< 0.12			< 2.50	< 0.12	4.34#	0.19
Target Valence	*1*,*19*	10.30**	0.35			4.70*	0.20	3.54#	0.16
Session 3: Semantic Relatedness x Target Valence	*1*,*19*	5.12*	0.21			4.50*	0.20	< 2.50	< 0.12
Target Valence	*1*,*19*	10.76**	0.36			8.59*	0.31	10.61**	0.36
Session 4: Semantic Relatedness x Target Valence	*1*,*19*	< 2.50	< 0.12			< 2.50	< 0.12	< 2.50	< 0.12
Target Valence	*1*,*19*	24.68***	0.36			< 2.50	< 0.12	< 2.50	< 0.12

Note

***p < .001

**p < .01

*p < .05

#*p* < .1

**Table 4:** The means and standard deviations of N1/P1 amplitude.

*FACTOR*		80–150 ms (N1/P1)			
		*Quadrant*		*Midline*	
		*mean (μV)*	*SD (μV)*	*mean (μV)*	*SD (μV)*
**Quadrant x Affective Congruence**		* *		* *	
Left Anterior	Affectively Congruent	-1.20	1.17		
	Affectively Incongruent	-1.40	1.50		
Right Anterior	Affectively Congruent	-1.20	1.20		
	Affectively Incongruent	-1.20	1.24		
Left Central	Affectively Congruent	-1.60	1.50		
	Affectively Incongruent	-1.53	1.70		
Right Central	Affectively Congruent	-1.21	1.56		
	Affectively Incongruent	-1.10	1.20		
Left Posterior	Affectively Congruent	0.10	2.00		
	Affectively Incongruent	0.42	1.72		
Right Posterior	Affectively Congruent	0.80	2.00		
	Affectively Incongruent	1.10	1.80		
**Semantic Relatedness**					
	Semantically Related	-1.00	1.20	-1.60	-1.80
	Semantically Unrelated	-0.40	1.10	-1.00	1.50
**Quadrant x Semantic Relatedness**					
Left Anterior	Semantically Related	-1.60	1.40		
	Semantically Unrelated	-1.00	1.30		
Right Anterior	Semantically Related	-1.50	1.30		
	Semantically Unrelated	-1.00	1.20		
Left Central	Semantically Related	1.80	1.70		
	Semantically Unrelated	-1.30	1.50		
Right Central	Semantically Related	-1.50	1.60		
	Semantically Unrelated	-0.80	1.40		
Left Posterior	Semantically Related	-0.01	2.00		
	Semantically Unrelated	0.40	2.00		
Right Posterior	Semantically Related	0.60	1.80		
	Semantically Unrelated	1.30	2.00		
**Target Valence**					
	Neutral	-0.50	1.10	-1.00	1.60
	Positive	-1.00	1.20	-1.60	1.70
**Quadrant x Target Valence**					
Left Anterior	Neutral	-0.10	1.30		
	Positive	-1.50	1.30		
Right Anterior	Neutral	-1.00	1.10		
	Positive	-1.40	1.20		
Left Central	Neutral	-1.30	1.50		
	Positive	-1.80	1.70		
Right Central	Neutral	-1.00	1.30		
	Positive	-1.50	1.30		
Left Posterior	Neutral	2.80	1.80		
	Positive	1.20	2.00		
Right Posterior	Neutral	1.10	1.80		
	Positive	-0.80	2.00		

**Table 5:** The means and standard deviations of P2/N2 amplitude.

*FACTOR*		150–300 ms (P2/N2)			
		*Quadrant*		*Midline*	
		*mean (μV)*	*SD (μV)*	*mean (μV)*	*SD (μV)*
**Semantic Relatedness**					
	Semantically Related	1.10	2.10	2.30	2.90
	Semantically Unrelated	1.50	2.20	2.80	2.90
**Session x Semantic Relatedness**					
Session 1	Semantically Related	1.00	2.20	2.20	3.00
	Semantically Unrelated	1.10	2.20	2.30	2.80
Session 2	Semantically Related	1.00	3.00	2.00	4.00
	Semantically Unrelated	1.80	2.80	3.20	3.60
Session 3	Semantically Related	0.90	2.00	2.20	2.70
	Semantically Unrelated	1.30	2.30	2.60	3.00
Session 4	Semantically Related	1.50	2.00	2.50	2.80
	Semantically Unrelated	2.00	2.00	3.00	2.90
**Quadrant x Semantic Relatedness**					
Left Anterior	Semantically Related	2.10	2.40		
	Semantically Unrelated	2.50	2.50		
Right Anterior	Semantically Related	1.40	2.20		
	Semantically Unrelated	1.90	2.20		
Left Central	Semantically Related	1.40	2.50		
	Semantically Unrelated	1.80	2.60		
Right Central	Semantically Related	1.00	2.30		
	Semantically Unrelated	1.50	2.30		
Left Posterior	Semantically Related	-0.01	2.60		
	Semantically Unrelated	0.30	2.70		
Right Posterior	Semantically Related	0.60	2.10		
	Semantically Unrelated	1.10	2.20		

**Table 6:** The means and standard deviations of N400 amplitude.

*FACTOR*		350–550 ms (N400)			
		*Quadrant*		*Midline*	
		*mean (μV)*	*SD (μV)*	*mean (μV)*	*SD (μV)*
**Session**					
	Session 1	2.10	2.70	2.50	3.00
	Session 2	3.40	3.50	4.20	4.40
	Session 3	4.00	3.10	4.50	3.80
	Session 4	3.70	2.70	4.20	3.80
**Target Valence**					
	Neutral	2.90	2.70	3.30	3.40
	Positive	3.70	2.90	4.40	3.70
**Session x Affective Congruence x Semantic Relatedness**					
Session 1	Congruent Related	2.24	2.82		
	Congruent Unrelated	1.86	3.10		
	Incongruent Related	2.16	2.64		
	Incongruent Unrelated	2.03	2.63		
Session 2	Congruent Related	3.51	3.75		
	Congruent Unrelated	3.52	3.74		
	Incongruent Related	3.27	3.52		
	Incongruent Unrelated	3.46	3.35		
Session 3	Congruent Related	4.00	3.10		
	Congruent Unrelated	3.94	3.44		
	Incongruent Related	4.25	3.10		
	Incongruent Unrelated	3.76	3.25		
Session 4	Congruent Related	4.10	2.60		
	Congruent Unrelated	3.30	3.04		
	Incongruent Related	3.47	2.73		
	Incongruent Unrelated	3.87	2.91		
**Affective Congruence x Target Valence**					
Congruent	Neutral	3.00	2.74		
	Positive	3.61	3.07		
Incongruent	Neutral	2.80	2.70		
	Positive	3.80	2.80		
**Session x Semantic Relatedness x Target Valence**					
Session 1	Neutral Related	1.97	2.30		
	Positive Related	2.44	2.89		
	Neutral Unrelated	1.65	2.44		
	Positive Unrelated	2.23	3.20		
Session 2	Neutral Related	3.26	3.49		
	Positive Related	3.52	3.71		
	Neutral Unrelated	3.10	3.64		
	Positive Unrelated	3.90	3.36		
Session 3	Neutral Related	3.39	2.73		
	Positive Related	4.86	3.27		
	Neutral Unrelated	3.58	3.27		
	Positive Unrelated	4.11	3.47		
Session 4	Neutral Related	3.34	2.73		
	Positive Related	4.22	2.66		
	Neutral Unrelated	2.91	3.01		
* *	Positive Unrelated	4.26	3.11		

**Table 7:** The means and standard deviations of LPC amplitude.

*FACTOR*		550–700 ms (LPC)			
		*Quadrant*		*Midline*	
		*mean (μV)*	*SD (μV)*	*mean (μV)*	*SD (μV)*
**Session**					
	Session 1	4.48	1.91	5.37	2.10
	Session 2	5.60	2.57	6.63	2.96
	Session 3	6.13	2.15	7.00	2.24
	Session 4	4.79	2.27	5.47	3.11
**Target Valence**					
	Neutral	5.02	1.79	5.85	2.04
	Positive	5.47	2.03	6.39	2.32
**Session x Affective Congruence x Semantic Relatedness**					
Session 1	Congruent Related	4.65	2.39	5.54	2.90
	Congruent Unrelated	4.53	2.36	5.49	2.55
	Incongruent Related	4.49	1.92	5.40	2.20
	Incongruent Unrelated	4.25	1.74	5.10	2.13
Session 2	Congruent Related	5.47	3.00	6.40	3.57
	Congruent Unrelated	5.61	2.95	6.74	3.38
	Incongruent Related	5.76	2.71	6.80	3.00
	Incongruent Unrelated	5.60	2.25	6.60	2.82
Session 3	Congruent Related	6.10	2.33	6.91	2.40
	Congruent Unrelated	6.44	2.58	7.34	2.85
	Incongruent Related	5.76	2.71	7.27	2.31
	Incongruent Unrelated	5.60	2.25	6.50	2.48
Session 4	Congruent Related	4.82	2.44	5.40	3.35
	Congruent Unrelated	4.40	2.53	4.98	3.45
	Incongruent Related	4.52	2.61	5.29	3.59
	Incongruent Unrelated	5.40	2.20	6.23	2.86
**Session x Semantic Relatedness x Target Valence**					
Session 1	Neutral Related	4.61	2.29	5.58	2.83
	Positive Related	4.53	1.98	5.33	2.25
	Neutral Unrelated	4.07	1.66	4.98	1.91
	Positive Unrelated	4.71	2.43	5.58	2.60
Session 2	Neutral Related	5.61	2.91	6.68	3.20
	Positive Related	5.62	2.82	6.51	3.38
	Neutral Unrelated	5.20	2.54	6.12	2.97
	Positive Unrelated	5.98	2.69	7.22	3.23
Session 3	Neutral Related	5.52	2.13	6.25	2.31
	Positive Related	6.83	2.65	7.92	2.74
	Neutral Unrelated	5.88	2.22	6.55	2.50
	Positive Unrelated	6.28	2.42	7.28	2.68
Session 4	Neutral Related	4.42	2.31	5.00	2.98
	Positive Related	4.93	2.87	5.69	4.13
	Neutral Unrelated	4.89	2.39	5.61	3.29
	Positive Unrelated	4.90	2.35	5.61	3.01

#### N1/P1 (80–150 ms)

Repeated measure ANOVAs for ERP amplitudes in the time-window 80–150 ms post-stimulus onset showed significant main effects of semantic relatedness. L2 targets preceded by semantically related L1 primes elicited more negative going-waves than those preceded by unrelated L1 primes. The effect of target valence was also significant. Positive L2 targets elicited more negative-going waves compared to neutral L2 targets.

**Fig 1:**
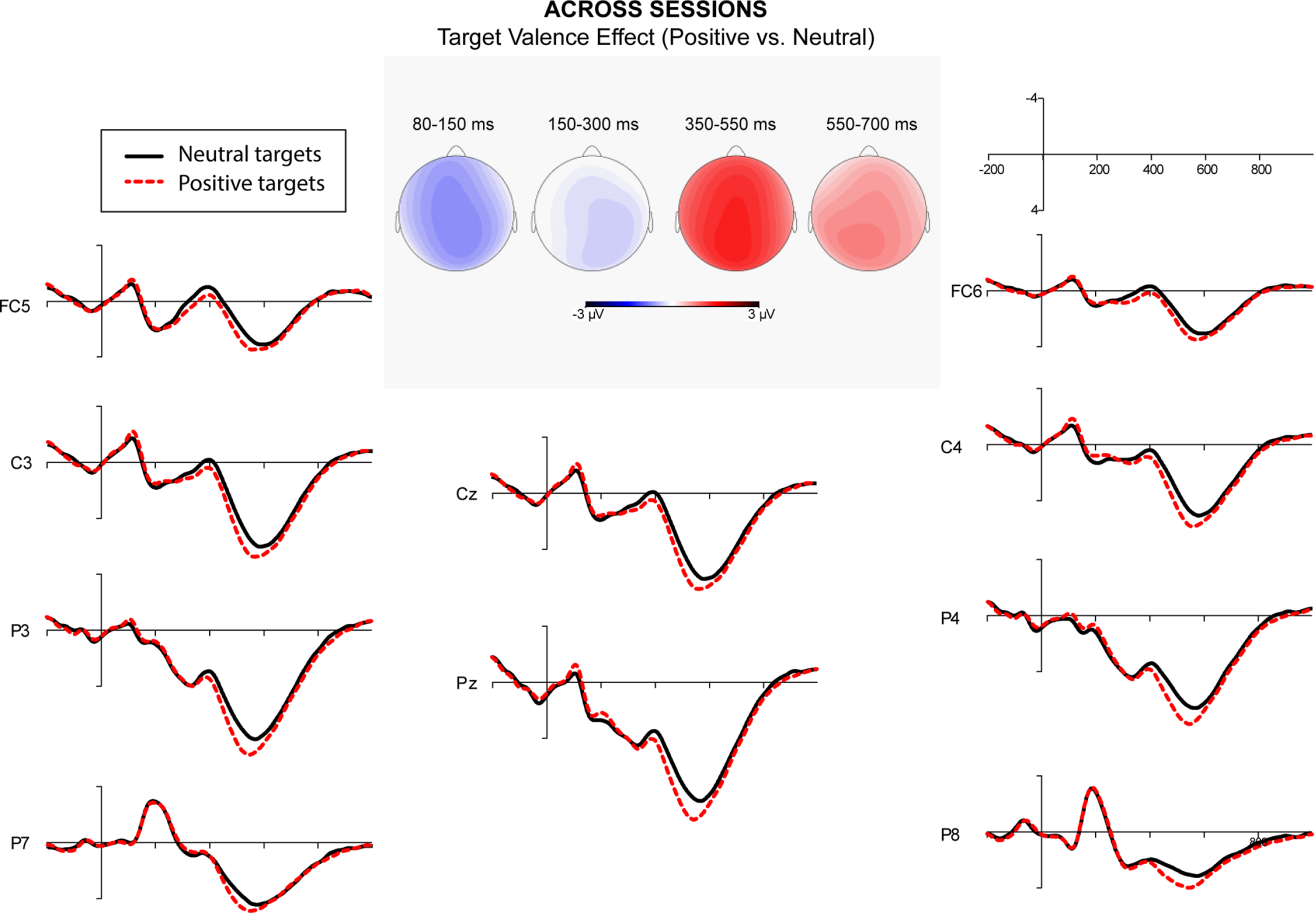
The ERPs in response to neutral and positive L2 targets across sessions (collapsing Session 1 to 4).

**Fig 2:**
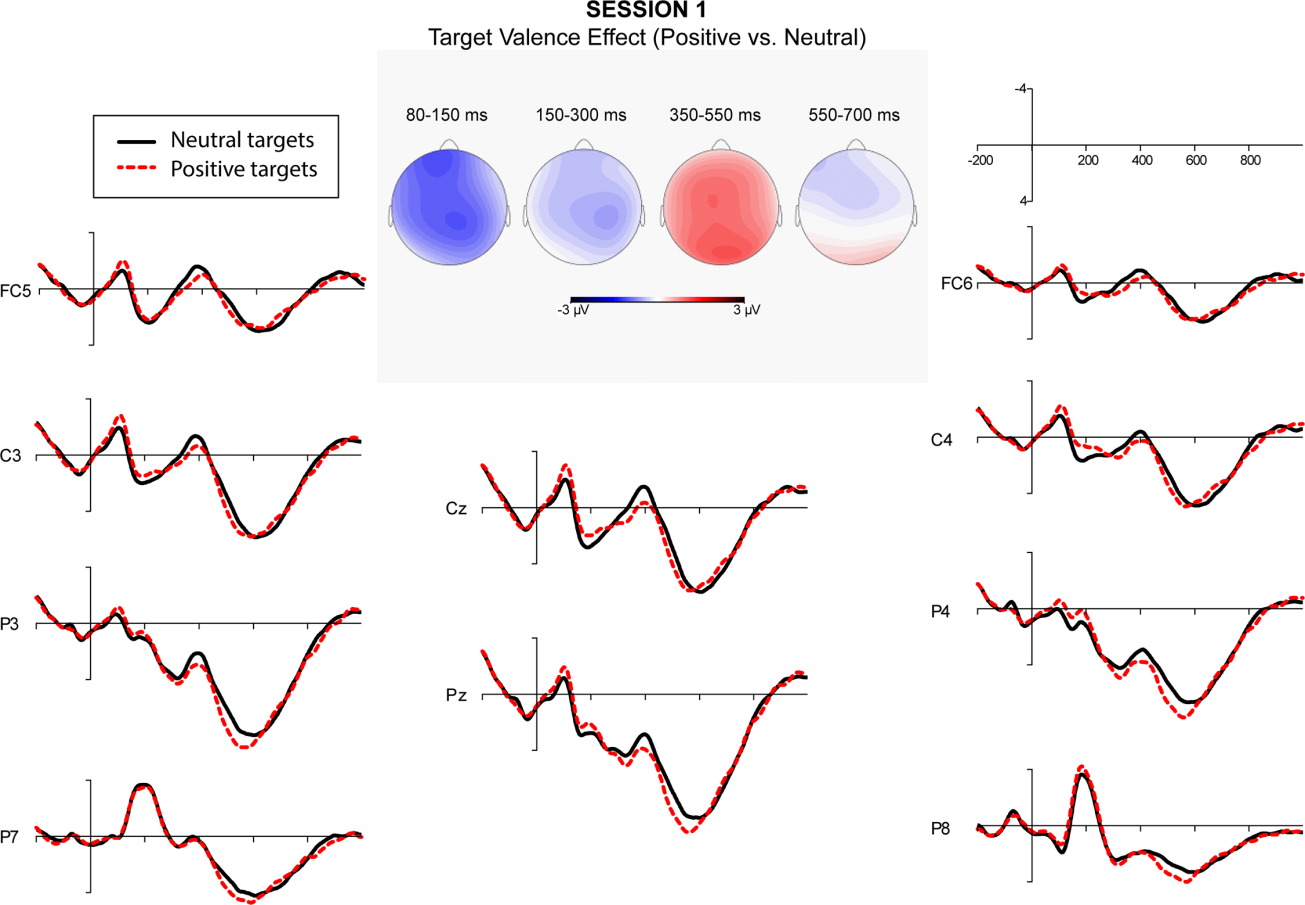
The ERPs in response to neutral and positive L2 targets in Session 1 (5–12 days after arrival).

**Fig 3:**
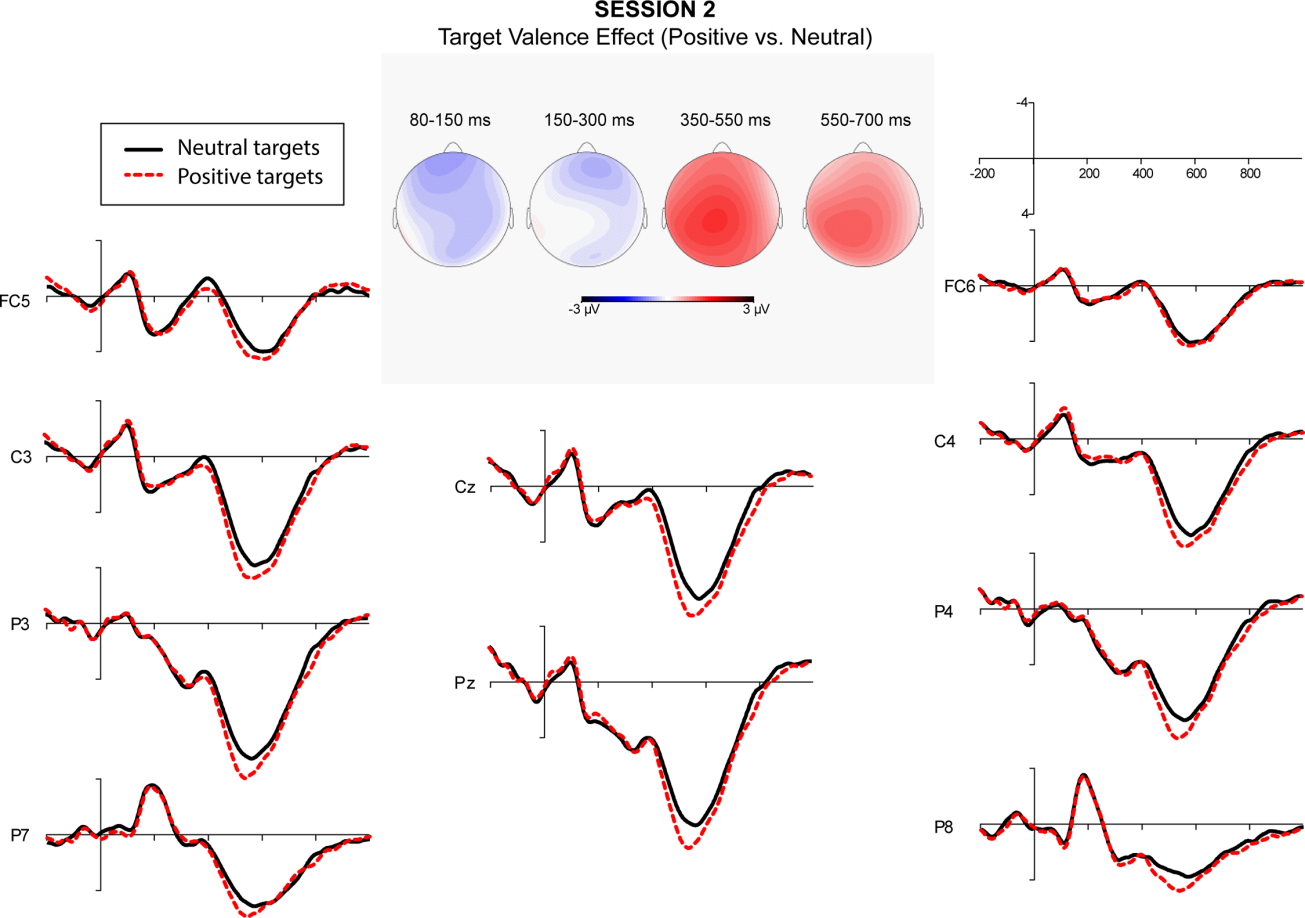
The ERPs in response to neutral and positive L2 targets in Session 2 (26–33 days after arrival).

**Fig 4:**
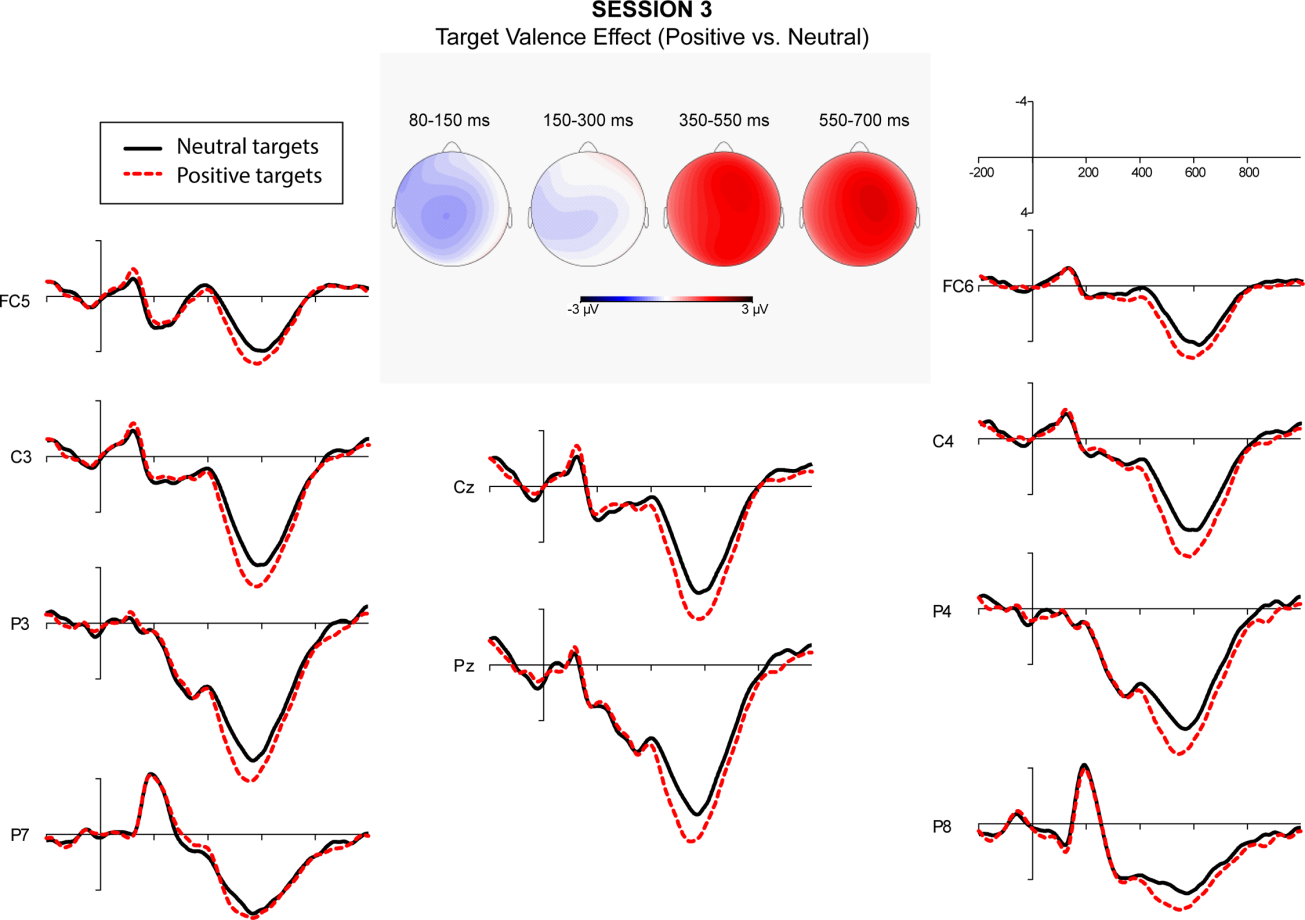
The ERPs in response to neutral and positive L2 targets in Session 3 (79–90 days after arrival).

**Fig 5:**
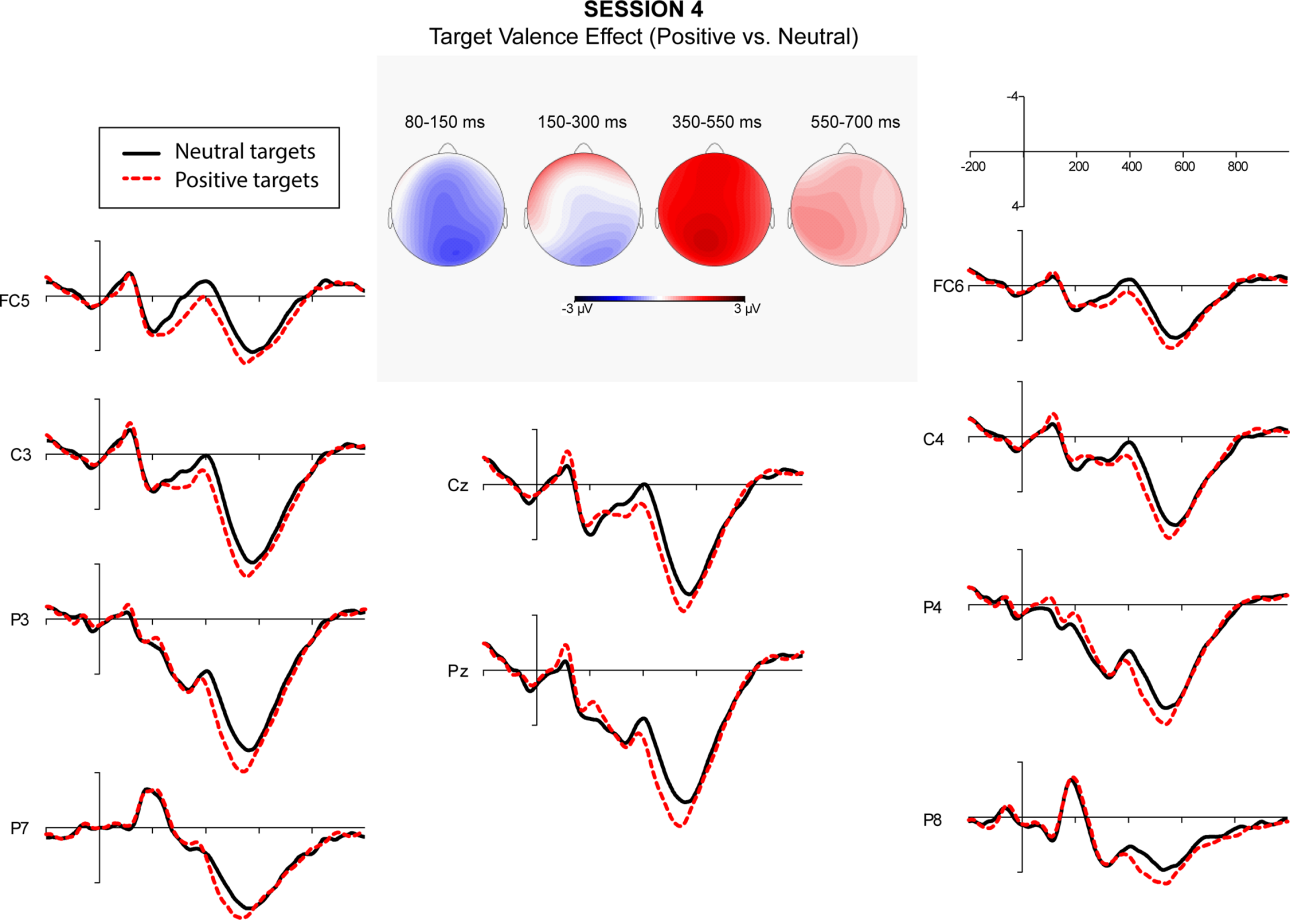
The ERPs in response to neutral and positive L2 targets in Session 4 (156–171 days after arrival).

A repeated measure ANOVA over quadrants showed a significant interaction between semantic relatedness and quadrant. Post-hoc comparisons indicated that semantic relatedness effects were significant at all quadrants. A significant interaction of affective congruence and quadrant was also obtained. Post-hoc comparisons showed that L2 targets preceded by affectively congruent L1 primes elicited a more negative-going wave than those preceded by affectively incongruent L1 primes at the right posterior quadrant. Finally, the interaction of target valence and quadrant was significant. Post-hoc comparisons showed that target valence effect was significant at all quadrants except the left posterior (Fig 1). Figs 2–5 showed the ERPs of Target Valence effect in every session.

To summarize, in the N1/P1 time window, strong overall effects were observed of semantic relatedness, both as a main effect and in interaction with quadrant. In addition, there were effects of target valence, again as a main effect and in interactions with quadrant. Finally, we observed an interaction between affective congruence and quadrant (see Tables 2 and 4 for details).

#### P2/N2 (150–300 ms)

Repeated measure ANOVAs over quadrant and midline areas showed significant main effects of semantic relatedness. L2 targets preceded by semantically unrelated L1 primes elicited more positive-going waves than those preceded by L1 semantically related primes. The interaction between semantic relatedness and quadrant was also significant. Post-hoc comparisons showed that the semantic priming effect was significant at all quadrants. Moreover, the interaction of session and semantic relatedness was also significant. Post-hoc repeated measure ANOVAs over the amplitudes of quadrant and midline electrodes did not show any significant semantic relatedness effects in Session 1. However, in Session 2, L2 targets preceded by semantically unrelated L1 primes elicited significantly more positive amplitudes than those preceded by related L1 primes. In Session 3, this semantic relatedness effect on the ERP amplitudes was significant only at the quadrants, wherein L2 targets preceded by semantically unrelated L1 primes also elicited more positive-going waves than those preceded by related L1 primes. In Session 4, the semantic relatedness effect was significant only at the midline area.

To summarize, in the P2/N2 time window, effects of semantic relatedness were found, both as a main effect and in interaction with quadrant and session. The effects occurred especially in Sessions 2 and 3, but also in Session 4 (midline) (see Tables 2 and 5 for details).

#### N400 (350–550 ms)

The repeated measure ANOVAs showed a significant effect of session in the time window 350–550 ms. Across conditions, Session 2 elicited more positive-going waves than Session 1. However, the amplitudes did neither differ significantly between Sessions 2 and 3, nor between Sessions 3 and 4. The ANOVAs further revealed significant main effects of target valence, wherein positive L2 targets elicited more positive-going waves than neutral L2 targets (Fig 1).

**Fig 6:**
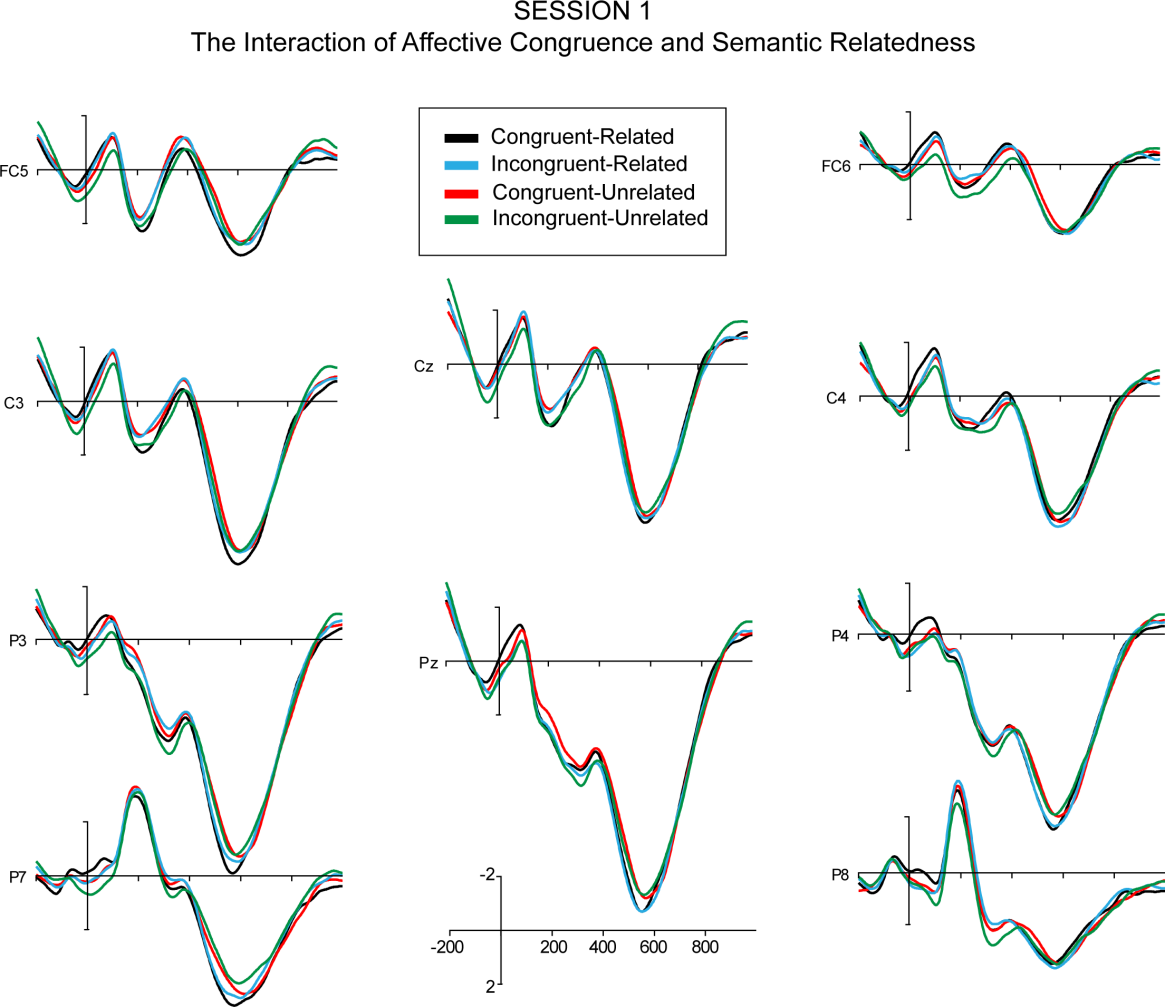
The ERPs of the interaction between Affective Congruence and Semantic Relatedness (time-locked to the onset of L2 targets) in Session 1.

**Fig 7:**
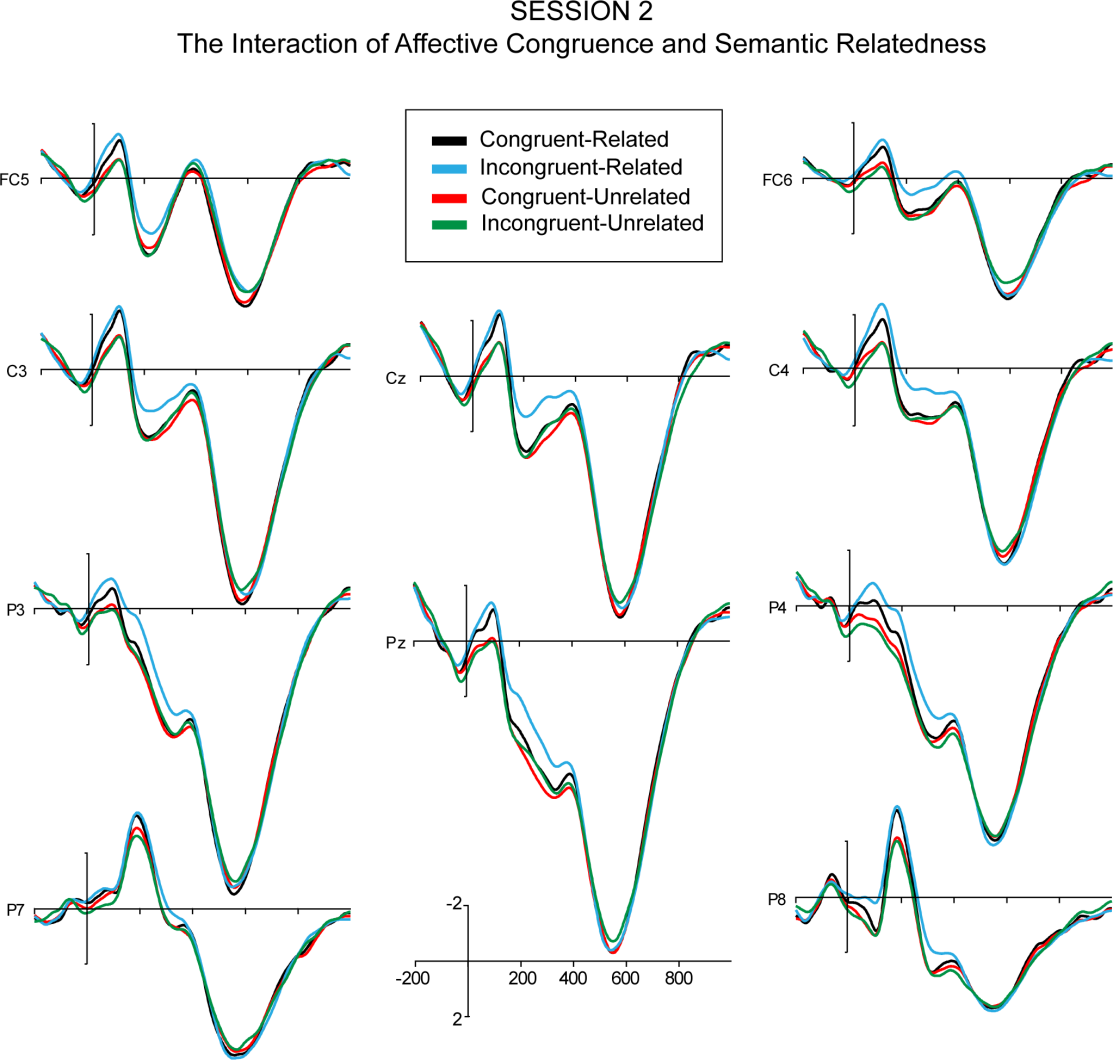
The ERPs of the interaction between Affective Congruence and Semantic Relatedness (time-locked to the onset of L2 targets) in Session 2.

**Fig 8:**
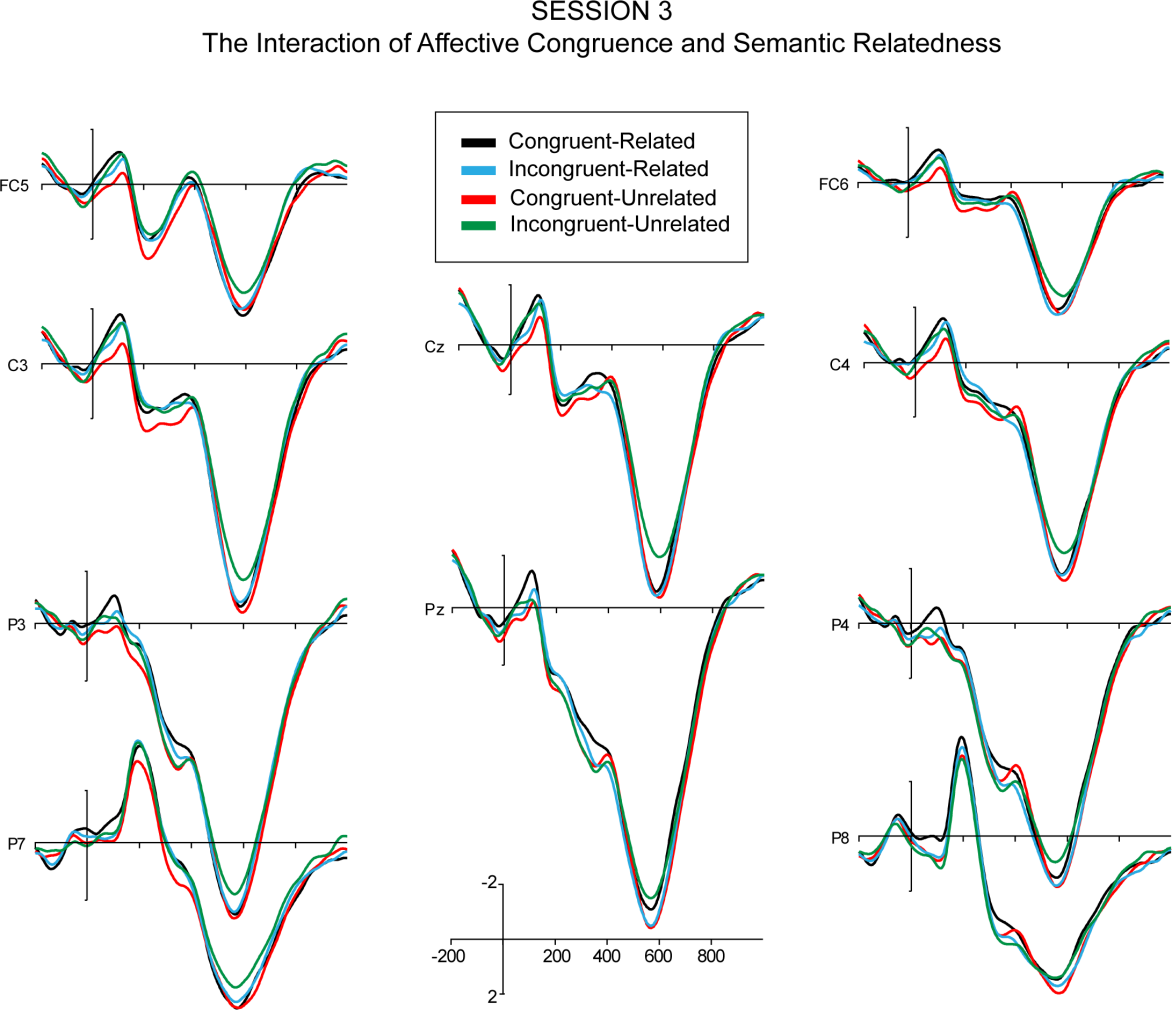
The ERPs of the interaction between Affective Congruence and Semantic Relatedness (time-locked to the onset of L2 targets) in Session 3.

**Fig 9:**
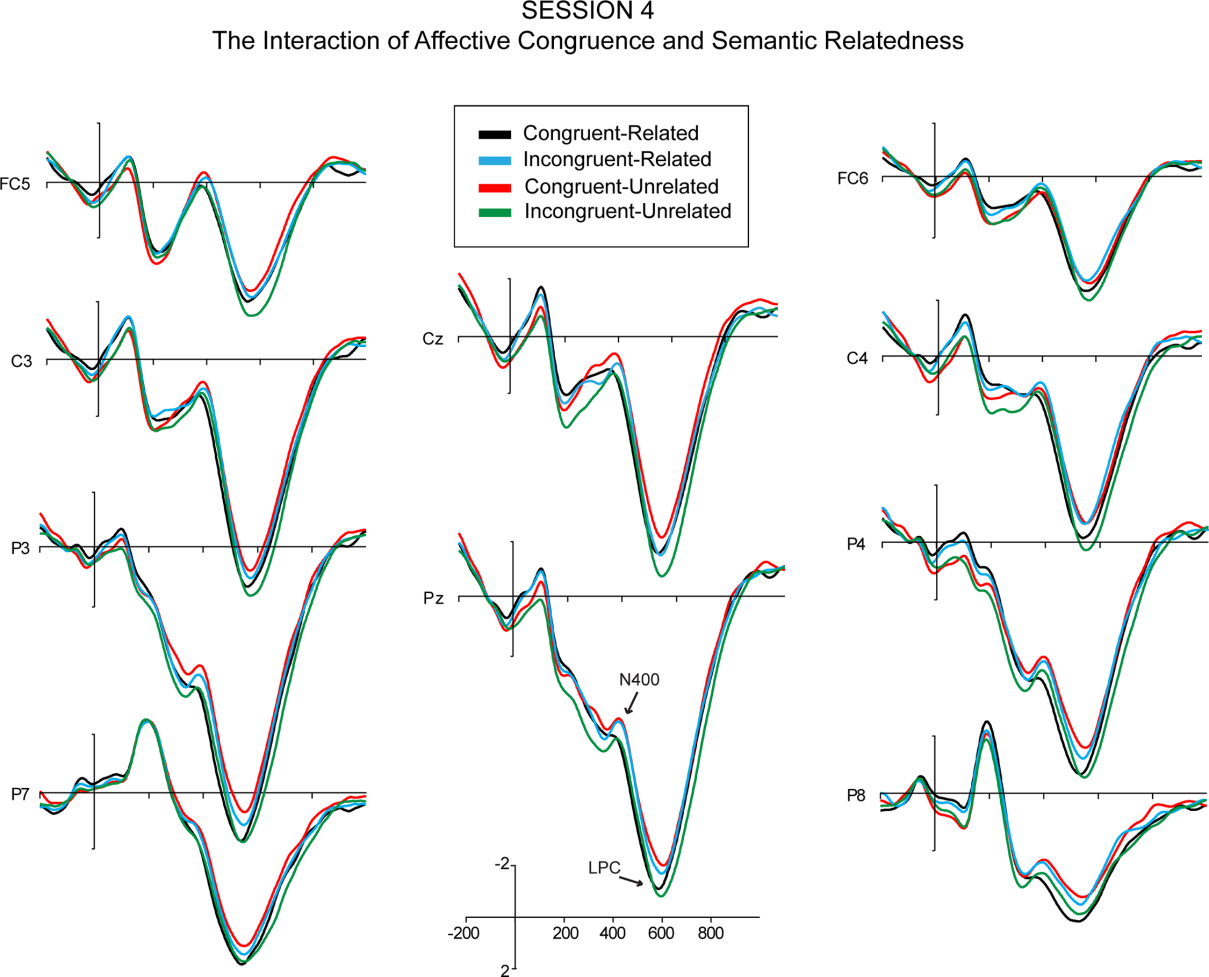
The ERPs of the interaction between Affective Congruence and Semantic Relatedness (time-locked to the onset of L2 targets) in Session 4.

The quadrant ANOVA revealed a significant 3-way interaction effect of session, affective congruence, and semantic relatedness, while in the midline ANOVA, the 3-way interaction effect was only marginally significant. For Sessions 1–3, post-hoc 2 (affective congruence) x 2 (semantic relatedness) repeated measure ANOVAs over amplitudes from the quadrant electrodes, showed no significant main effects of affective congruence or semantic relatedness and no significant interaction effect of affective congruence and semantic relatedness (Figs 6–8). However, the post-hoc ANOVA for Session 4 (Fig 9) showed a significant interaction between affective congruence and semantic relatedness, while no main effects were significant.

**Fig 10:**
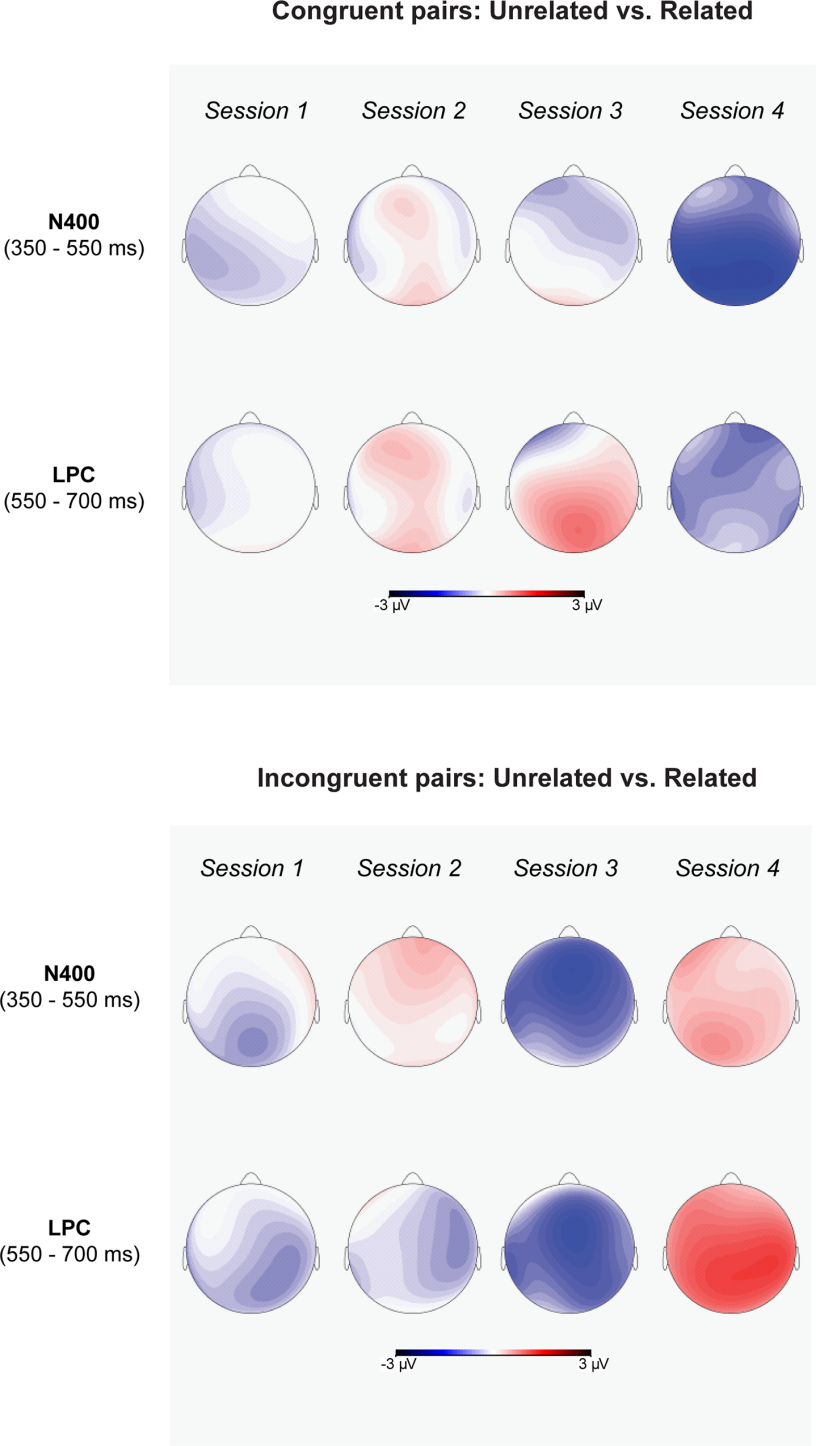
The scalp topography of semantic priming effects (after subtracting the ERP amplitudes for Related pairs from those for Unrelated pairs) in N400 and LPC time-windows for each session.

**Fig 11:**
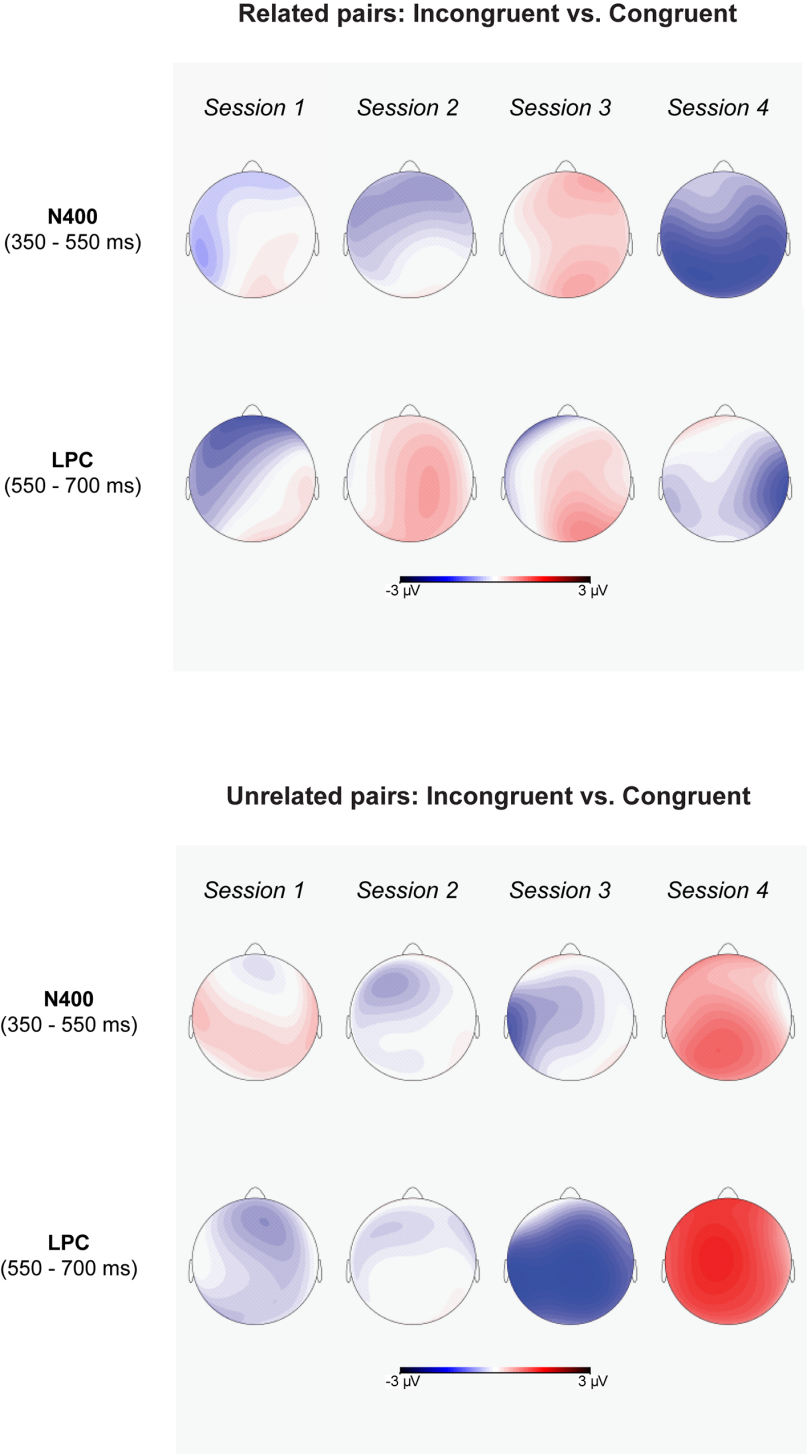
The scalp topography of affective priming effects (after subtracting the ERP amplitudes for Congruent pairs from those for Incongruent pairs) in N400 and LPC time-windows for each session.

Additional post-hoc pairwise comparisons showed a significant semantic relatedness effect for L2 targets preceded by affectively congruent L1 primes (Fig 10). More positive-going waves were elicited when the primes were also related to the targets than when the primes were unrelated to the targets (Fig 10). However, the ERP amplitude elicited by L2 targets preceded by affectively incongruent L1 primes was not significantly modulated by semantic relatedness. In addition, more post-hoc comparisons showed that L2 targets preceded by semantically related L1 primes elicited more positive going-waves when the primes were congruent than when the primes where incongruent to the targets (Fig 11). On the contrary, for L2 targets that were preceded by semantically unrelated L1 primes, the targets elicited more positive-going waves when the primes were also incongruent than when they were congruent to the targets (Fig 11).

In addition, the quadrant ANOVA revealed a significant 3-way interaction of session, semantic relatedness, and target valence. Post-hoc repeated measure ANOVAs revealed no significant main or interaction effects in Session 1; however, in Session 2, there was already a main effect of target valence, although the main effect of semantic relatedness and the interaction between semantic relatedness and target valence were not significant. In Session 3, the post-hoc ANOVA did not reveal any significant main effects, but the interaction between semantic relatedness and target valence was significant. More post-hoc pairwise comparisons showed that L2 targets preceded by semantically related L1 primes yielded more positive-going waves when the targets were positive than when they were neutral. However, a post-hoc ANOVA revealed no significant target valence effect for L2 targets preceded by semantically unrelated L1 primes. In Session 4, the main effect of semantic relatedness and the interaction effect of semantic relatedness by target valence were not significant. The post-hoc ANOVA also showed that the main effect of target valence was significant, wherein positive targets elicited more positive-going waves compared to neutral targets.

From the quadrant ANOVA, there was also a significant interaction between affective congruence and target valence. Post-hoc comparisons showed that positive L2 targets elicited more positive-going waves than neutral L2 targets.

To summarize, in the N400 window, many significant effects occurred within and across sessions. There was an overall main effect of target valence (see Fig 1 for Target Valence effect on N400 across sessions) and an interaction of target valence with affective congruence. Significant interactions of target valence, semantic relatedness, and session were obtained persistently over the four sessions. Finally, interactions of semantic relatedness, affective congruence, and session were observed that were especially strong in Session 4 (see Tables 3 and 6 for details).

#### LPC (550–700 ms)

Repeated measure ANOVAs showed a significant effect of session. Post-hoc comparisons showed that Session 2 elicited more positive-going waves compared to Session 1. The ERP amplitudes of Session 2 and 3 did not differ significantly. However, the difference between the ERP amplitudes of Sessions 3 and 4 was significant. There were also significant main effects of Target Valence, in which positive L2 targets yielded more positive-going waves than did neutral L2 targets (Fig 1).

Moreover, the ANOVAs revealed a significant 3-way interaction of session, affective congruence, and semantic relatedness. In Sessions 1 and 2, post-hoc ANOVAs revealed no significant main effects or interaction effects (Figs 6 and 7). In Session 3 (Fig 8), there were only marginally significant interaction effects of affective congruence and semantic relatedness. In Session 4 (Fig 9), there were no significant main effects either, but the interaction of affective congruence and semantic relatedness reached significance. Post-hoc pairwise comparisons showed affective congruence effects only for L2 targets preceded by semantically unrelated L1 primes, wherein targets preceded by affectively incongruent primes elicited more positive-going waves than those preceded by affectively congruent primes (Fig 11). Additional post-hoc comparisons showed that semantic relatedness effects occurred only for L2 targets preceded by affectively incongruent L1 primes, with L2 targets preceded by semantically unrelated primes eliciting larger positive-going waves than those preceded by semantically related primes (Fig 10).

The interaction effect between session, semantic relatedness, and target valence was also significant. Post-hoc ANOVAs revealed no significant main effects or interaction effects in Session 1. In Session 2, there was only a main effect of target valence, in which positive L2 targets elicited more positive-going waves than neutral L2 targets did. The interaction of semantic relatedness and target valence was not significant. In Session 3, there was no significant main effect of semantic relatedness, although the main effect of target valence was significant. In this session, positive L2 targets also elicited more positive-going waves than did neutral L2 targets. The interaction between semantic relatedness and target valence was significant. Post-hoc pairwise comparisons showed that positive L2 targets elicited more positive-going waves than neutral L2 targets only when they were preceded by semantically related L1 primes. However, post-hoc ANOVAs for Session 4 revealed no significant main effects or interaction effects.

To summarize, in the LPC time window, several effects occurred. Apart from a main effect of target valence, there were persistent significant interactions of target valence, semantic relatedness, and session that were especially strong in Sessions 2 and 3. Finally, interactions of semantic relatedness, affective congruence, and session were observed that were especially due to Session 4 (see Tables 3 and 7 for details).

## Discussion

We investigated how the development of L2 emotion-laden words during L2 learning is affected by L1 semantic properties. German (L1) learners of Dutch (L2) participated in an L1-to-L2 priming study with lexical decision, ranging over four sessions. The sessions started during a 5-week Dutch course the learners followed after their arrival in the Netherlands, and lasted to 6 months after their arrival when they were immersed in Dutch daily life. Focusing on the learning of L2 positive and neutral words, we manipulated both the semantic relatedness and affective congruence between L1 primes and L2 targets. We established the efficacy of L2 teaching on the basis of accuracy and RT measures (see S1 Text).

We will now summarize and discuss (especially) the EEG results relevant to the three aims we specified in the Introduction: (1) To determine the semantic and affective interconnectedness between L1 and L2 words; (2) To assess valence effects of L2 target words during L2 learning; (3) To pinpoint the interaction of semantic and affective meaning in L2 learning and processing.

### Semantic and affective interconnectedness between L1 and L2 words

First, we observed an overall small but significant *semantic priming effect* in accuracy rates and RTs across sessions (collapsing Sessions 1 to 4). These semantic priming effects were more prominent in the EEG, where we observed a main effect of semantic relatedness that modulated the N1/P1 time-window (80–150 ms). In the subsequent P2 time-window (150–300 ms), the semantic priming effect also modulated ERP amplitudes, both as a main effect and in interaction with session. This semantic effect in the early time-windows is in line with evidence from L1 priming studies that argue in favor of a rapid interaction of sublexical and lexical-semantic processing [40–42], presumably due to top-down feedback [43] from the L1 prime’s semantic activation. In other words, across sessions, there was a persistent effect of semantic relatedness on L2 lexical processing and semantic accessibility already in an early stage of target processing [24,44]. With regard to the interaction between session and semantic relatedness in our longitudinal study, L2 targets following related L1 primes consistently showed reduced P2 amplitudes starting from Session 2 onwards. This finding might be linked to a decrease in semantic processing demands [24] relative to targets following unrelated L1 primes. These results provide evidence for an interconnectedness of lexical and semantic networks across L1 and L2 neutral and emotion-laden words during early stages of L2 learning.

With respect to *affective priming*, subjects responded faster to congruent targets across sessions, although there was no significant effect of affective priming on the accuracy rates. Importantly, a consistent target valence effect was observed: Accuracy to positive targets was constantly higher than to neutral target across sessions, indicating that accessibility to L2 representations was reliably affected by the emotionality of L2 words, although there were no RT differences. For the EEG, we found an affective priming effect on the right posterior P1 component showing more reduced target’s P1 amplitude for congruent pairs than incongruent primes. Enhancement of P1 amplitude in affective priming paradigm may reflect the allocation of more early attention resources in the case of negatively-valenced, high-arousal primes (relative to low-arousal, positive and/or neutral ones) to capture attention [45]. Thus, the attentional ‘grabbing power’ of negative L1 primes employed in this study apparently enhanced the early perception of the target words. Altogether, our findings suggest that the L2 learners are sensitive to congruence and incongruence in the affective connotations of prime and target very early in L2 learning, and have stored the emotional quality of their L2 words in their lexical memory across the sessions. These results provide evidence for an interconnectedness of affective representations across L1 and L2 words.

### Valence effects of L2 target words during L2 learning

The generality of affective priming effects was backed up by the significant main effects of L2 Target Valence across the sessions on N1/P1 (80–150 ms), N400 (350–550 ms) and LPC time-windows (550–700 ms). These results indicated that the L2 learners really attended to affective aspects of L2 targets after 1 week of L2 learning. This is evidence for early onset of L2 valence-specific processing in adult learners.

Our findings on early valence processing in L2 are in line with the available literature in L1. Several L1 studies suggest that valence information in verbal stimuli may already be extracted in a very early stage of word processing, even before 100 ms [19,21,22]. According to Skrandies [20], an early valence effect in L1 is not surprising, because reading is a rapid process and, thus, the effect of semantic features such as valence might have already occurred during a primary visual analysis or early perceptual stage. One might perhaps not expect such a rapid process of extracting emotional information to occur in L2. However, in the present study, we incorporated L1 words as primes, which is the dominant language of the learners. Our results on affective priming effects (see Table 6) suggest that L1 priming modulated the time-course of the activation of words’ affective connotations [18] in L2, leading to activation of affective representation of L2 words in a very early stage of target processing. Seen in this way, our findings extend evidence of the early valence effect in L1 to L2 word processing through priming in L2 learners.

### Coupling of semantic and affective meaning in L2 learning and processing

In contrast to Castner et al. [9], we did not find a mutual modulation of the effects of Affective Congruence and Semantic Relatedness in accuracy rates or RTs. We therefore considered the ERPs, as this measure would be more sensitive to the time course and their differences in the consecutive stages of word processing. Indeed, here the semantic and affective properties of L1 and L2 interacted, as indicated by the interaction between Session, Affective Congruence, and Semantic Relatedness. However, only in Session 4 cross-language semantic and affective priming modulated the two late ERP components (N400 and LPC) altogether. Importantly, these results suggest learning-related changes in the link between semantic and affective representations, established when the learners have already been immersed in L2 environment to follow university courses in their L2. With respect to the N400, for incongruent and unrelated pairs, there was a more positive N400 compared to congruent-unrelated pairs. However, there was an even larger facilitation effect due to affective congruence in the related pairs, with congruent and related pairs exhibiting reduced N400 compared to incongruent-related pairs. The semantic priming effect was evident in the congruent pairs: For congruent and related pairs, there was a more positive N400 than for congruent unrelated pairs. This finding indicates that the learners were sensitive to differences in L1 and L2 meanings of both semantic and affective nature after 5 months of living in L2 environment.

Targets that mismatch with the primes in any of these aspects were harder to process: The inhibitory effects of unrelated L1 primes were significantly *attenuated* when these were also incongruent with respect to the affective connotations of L2 targets. This finding suggests that L1 negative primes may not always inhibit the processing of positive and neutral words, as claimed by a previous study on L1 priming [46]. Clearly, our L1-L2 priming results indicate that L1 negative primes do not prohibit the semantic integration of L2 words as long as their meanings do not have any associativeness to the meanings of L2 words. Conflicting affective information results in a reversed L1-L2 affective priming effect on semantic integration phase when the semantic aspect of the word meanings are also not associated with the prime in one condition but associated in another.

**Fig 12:**
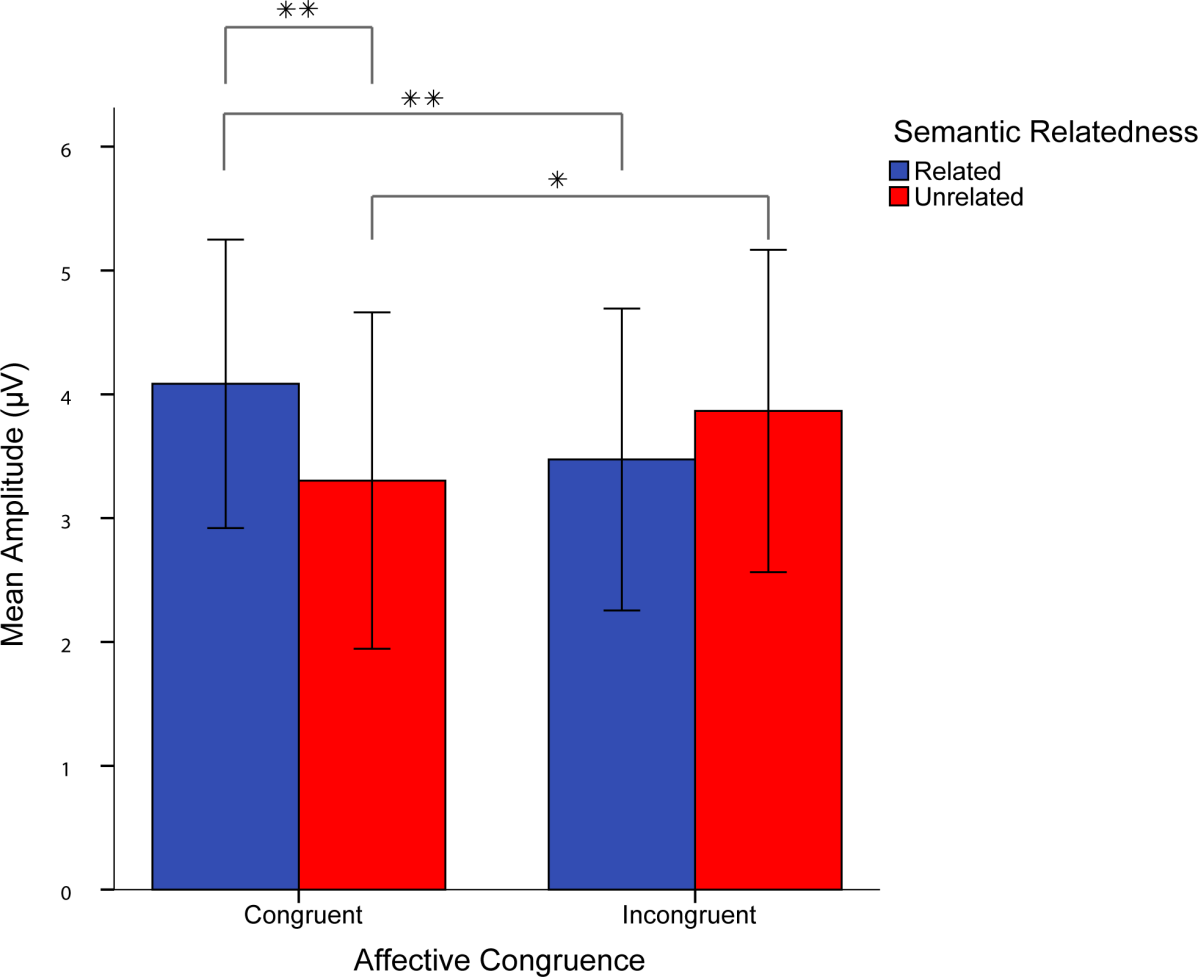
Mean amplitude (μV) of the semantic-affective interaction effect on N400 response in Session 4. Error bars represent standard error of the means.

In addition, positive and neutral L1 primes might not be easily integrated with L2 words with congruent emotional quality, especially when these primes do not have any meaning associations to the targets. On the other hand, the N400 effect of affectively congruent L1 primes was significantly larger when they were related rather than unrelated to the L2 targets. Moreover, the N400 semantic priming effect was expected to be reversed for affectively incongruent pairs, because we predicted that primes that mismatch on one dimension with the target would interfere with target processing. However, we note that the semantic priming effect was absent in the incongruent pairs: No difference was found between N400 amplitudes for unrelated and related pairs (see Fig 12). One explanation for this non-significant reversed N400 effect is that the negative aspect of the primes activates a compensatory mechanism that inhibits the spread of activation to related concepts [11,39,46].

With respect to the LPC, the L2 targets following L1 incongruent primes elicited more positive LPC amplitude when prime and targets were unrelated rather than related. The LPC for targets following L1 unrelated primes was also more positive when prime and targets were incongruent rather than congruent. Because the amplitude of LPC indexes increased attention and the depth of stimuli evaluative processing in the affective priming paradigm [26,27,47], this late positive-going ERP component may exhibit a larger amplitude when primes and targets are incongruent, suggesting that more motivated attention was exerted due to enhanced processing of target evaluation. Thus, our findings suggest that the LPC effects on L2 targets were modulated by both the affective incongruence between L1 primes and L2 targets, and their semantic relatedness. The more positive LPC amplitude for incongruent-unrelated pairs than for both incongruent-related and congruent-unrelated pairs suggests increased engagement of attention to process positive and neutral L2 targets following negative L1 primes without meaning association to the targets. Note that our LPC interaction effects are in line with findings from Castner et al. [9], who showed a semantic facilitation effect of L1 within-language priming in incongruent pairs and an affective facilitation effect in unrelated pairs. Our longitudinal study shows an interaction between L1-L2 semantic and affective priming in N400 and LPC time-windows that became stronger in the later immersion period, when L2 learners were highly exposed to an L2 social environment.

### Limitations of the study

As for any study, we need to consider potential limitations to our generalization of the conclusions. First of all, we investigated German learners of Dutch to see how early valence and affective congruence effects would arise in these similar languages, in which a number of form and meaning related words (so-called cognates) exist [48]. Many other similar language pairs exist in Europe, for instance, Danish and Norwegian, and Italian and Spanish. We investigated the general linkage of semantic and affective representations in L1 and L2, which is likely to be independent of the specificities of translation pairs. Therefore, we do not think that the choice of this combination of languages limits our conclusions. Furthermore, the participants, representatives of German college students in the Netherlands, also shared much of their cultural background with Dutch college students [49]. Cultural differences might also contribute to differences in valence qualities with respect to semantic representations. In all, it would therefore be of interest to future studies, first, to clarify the nature of affective properties in cognates, and second, to consider the interaction of semantic and affective priming in language learning by learners from cultures and languages that are very different, for instance, Chinese learners of Dutch.

A second issue to consider is that a significant interaction of Session, Affective Congruence, and Semantic Relatedness was found only in the ERP analyses. Because ERPs are often considered to be more sensitive than behavioral measures, the lack of a significant interaction effect in the RTs might reflect a power problem. Future studies should take this into consideration when they elaborate and extend the results of our study.

A third issue concerns the relationship between valence and arousal properties of words. When we prepared our stimulus materials, it turned out to be impossible to equate negative, neutral, and positive words in terms of arousal properties. As a consequence, our negative German L1 primes were always higher in arousal value than our positive and neutral German L1 primes. Thus, a contribution of arousal to the present results cannot be excluded. This is a methodological point that is applicable to several of the studies in this domain [9,50,51]. It could be that the general arousal levels of emotional stimulus materials used in different studies are not equal and interact with valence congruence effects in affective priming [50]. If this were the case, it would account for the different or even opposite results that studies have reported.

As a fourth issue, the complexity of this domain forced us to include in our study only positive and neutral targets, whereas some other studies focused on negative and neutral [9], or negative and positive items [7,8]. In addition, we limited our study to L1-L2 priming and included no priming in the opposite direction. It is possible, even likely, that priming from L2 to L1 would yield in a different pattern of results. Clearly, these restrictions put some limits on the conclusions and generalizability of studies, including ours.

### Conclusion

We found various semantic and affective priming effects on behavioral and ERP measures of L2 word processing in different L2 learning stages. Non-interacting semantic and affective priming effects in early learning stages indicate the existence of separate representations for semantic and affective aspects of word meaning that themselves are shared across L1 and L2. Already in the earliest stage of L2 learning, a valence-specific effect on early and late ERPs was observed. Because studies like ours also show that bilingual language processing is influenced by the valence of words, this finding must be accounted for in models like BIA+ [52,53], Revised Hierarchical Model [54], or Inhibitory Control [55,56]. A coupling of cross-language affective and semantic processing was found to arise more gradually, dependent on session. The obtained affective-semantic interaction over sessions within N400 and LPC time-windows points at learning-related changes in the linkage of semantic and affective processing in L2 learning. In all, our ERP findings reflect the developing interactive network of lexical meaning representations in unbalanced L2 learners. The applied longitudinal and electrophysiological approach to L2 word learning thus provides a new and useful tool to understanding when and how a new language starts to be processed not only semantically but also affectively during standard class-room teaching practice.

## Supporting Information

S1 Dataset (ZIP)

S1 Table (DOCX)

S1 Text (DOCX)

S2 Text (DOCX)
