# Supplementary material for: When Feelings Arise with Meanings: How Emotion and Meaning of a Native Language Affect Second Language Processing in Adult Learners
Source: PLoS One. 2015 Dec 10;10(12):e0144576. doi: 10.1371/journal.pone.0144576 (PMC4684350; doi:10.1371/journal.pone.0144576)
Supplement: S1 Table — (DOCX) [file pone.0144576.s002.docx]

# S1 Table. Stimuli list and characteristics

| **S1A Table. The list of stimuli.** | |  |  |  |
| --- | --- | --- | --- | --- |
| **L1 PRIMES** | | | | ***L2 Neutral Targets*** |
| Related | | Unrelated | |  |
| Congruent | Incongruent | Congruent | Incongruent |  |
| Frage (question) | Problem (problem) | Adressat (addressee) | Schande (shame) | antwoord (answer) |
| Mitglied (member) | trennen (separate) | Tabak (tobacco) | Gauner (crook) | deel (part) |
| Schatten (shadow) | Angst (fear) | Besen (broom) | Marter (torture) | donker (dark) |
| Dose (can) | Gift (poison) | Abtei (abbey) | Folter (torture) | fles (bottle) |
| Neubau (new construction) | Kollaps (collapse) | Ausgabe (issue) | Arroganz (arrogance) | gebouw (building) |
| Erzeugnis (product) | Sucht (addiction) | Aufzucht (rearing) | gruselig (spooky) | gebruik (use) |
| Nachbar (neighbour) | Miete (rent) | Abdruck (impression) | voreilig (rash) | inwoner (resident) |
| Mauer (wall) | Wecker (alarm clock) | Borste (bristle) | Klaue (claw) | klok (clock) |
| Geruch (odor) | Sarg (coffin) | Klima (climate) | Panzer (tank) | lichaam (body) |
| Woche (week) | Zahlung (payment) | Inhalt (contents) | Nachruf (obituary) | maand (month) |
| Marke (brand) | gemein (common) | Mittel (resources) | Geiz (stinginess) | naam (name) |
| Tisch (table) | Putzen (clean) | Punkt (point) | Monstrum (monster) | plaat (plate) |
| Haube (hood) | knebeln (gag) | Rudel (pack) | Beil (Ax) | sjaal (scarf) |
| Fahrer (driver) | Stau (congestion) | Neugier (curiosity) | Bahre (stretcher) | straat (street) |
| Bohrer (drill) | Schmerz (pain) | Versteck (hiding place) | Raub (robbery) | tandarts (dentist) |
| Stunde (hour) | Frist (deadline) | Thema (topic) | Soldat (soldier) | tijd (time) |
| Aal (eel) | Wurm (worm) | Leim (glue) | Unfall (accident) | vis (fish) |
| Sohle (zool) | Fessel (shackle) | Achse (axis) | Steuer (tax) | voet (foot) |
| Laken (sheet) | Mumie (mummy) | Orden (order) | Rakete (rocket) | wit (white) |
| Kohle (coal) | Trauer (mourning) | Fahne (flag) | Tyrann (tyrant) | zwart (black) |
|  |  |  |  |  |
| **S1A Table-continued.** |  |  |  |  |
| **L1 PRIMES** | | | | ***L2 Positive Targets*** |
| Related | | Unrelated | |  |
| Congruent | Incongruent | Congruent | Incongruent |  |
| sonnig (sunny) | Regentag (rainy day) | Veilchen (violet) | Träne (tear) | daglicht (daylight) |
| tanzen (dance) | Inferno (Hell) | Urwald (jungle) | Schrott (scrap) | feestje (party) |
| froh (happy) | traurig (sad) | Weite (width) | bankrott (bankrupt) | gelukkig (happy) |
| Medizin (medicine) | Krankheit (disease) | Euphorie (euphoria) | Schlinge (loop) | gezondheid (health) |
| Tugend (virtue) | Kontrolle (control) | vertraut (familiar) | Fraktion (fraction) | kwaliteit (quality) |
| Herbst (autumn) | Wanze (bug) | Einheit (unit) | allein (alone) | lente (spring) |
| Erfahrung (experience) | sterben (die) | Erwerb (acquisition) | Kanone (cannon) | leven (life) |
| reizvoll (attractive) | scheu (shy) | Stärke (strength) | Kanal (channel) | meisje (girl) |
| Fähigkeit (talent) | Unheil (disaster) | Vorsorge (provision) | Miliz (militia) | natuurlijk (natural) |
| Komfort (comfort) | schlimm (bad) | dank (thanks) | stinken (stink) | prettig (nice) |
| Bett (bed) | Tod (death) | Segel (sail) | Bombe (bomb) | slapen (sleep) |
| süß (sweet) | sauer (sour) | Lohn (wage) | Natter (snake) | smaak (taste) |
| Familie (family) | Abschied (farewell) | Magier (magician) | Narkose (anesthesia) | thuis (home) |
| wachsen (grow) | Unkraut (weed) | Seide (silk) | Kanzler (chancellor) | tuin (garden) |
| brillant (brilliant) | protzen (proud) | Anregung (stimulation) | Gejammer (whining) | uitstekend (excellent) |
| sichern (secure) | Gefahr (danger) | Aroma (aroma) | Makel (stigma) | veilig (safe) |
| Geschenk (gift) | altklug (precocious) | Befreiung (exemption) | Übel (evil) | verjaardag (birthday) |
| Friede (peace) | Krise (crisis) | Kamin (fireplace) | Narbe (scar) | wereld (world) |
| Einigung (agreement) | Zweifel (doubt) | Fantasie (fantasy) | Tadel (scar | zeker (certainly) |
| Mond (moon) | Hitze (heat) | Mahl (meal) | Darm (intestine) | zon (sun) |
|  |  |  |  |  |

| **S1B Table. The list of stimuli characteristics.** | | | | | |
| --- | --- | --- | --- | --- | --- |
| **PRIMES** | | | | | |
| Prime types | Valence | Arousal | Imageability | Frequency (SUBTLEX-DE) | Length |
| Negative | -1.70 (0.60) | 3.40 (0.71) | 4.20 (1.19) | 2.14 (0.81) | 6.30 (1.40) |
| Positive | 1.50 (0.60) | 2.40 (0.72) | 4.50 (1.19) | 2.32 (0.84) | 6.30 (1.40) |
| Neutral | -0.01 (0.19) | 2.40 (0.73) | 4.30 (1.19) | 2.13 (0.81) | 6.10 (1.30) |
|  |  |  |  |  |  |
| **TARGETS** | | | | | |
| Target types | Valence | Arousal | Imageability | Frequency (SUBTLEX-NL) | Length |
| Positive | 7.90 (0.70) | 5.50 (1.50) | 7.10 (1.70) | 3.60 (0.70) | 6.80 (2.00) |
| Neutral | 5.60 (0.50) | 4.00 (1.00) | 6.70 (2.00) | 3.40 (0.60) | 5.30 (1.60) |
|  |  |  |  |  |  |

| **S1C Table. List of Pseudoword targets** | | | |  |  |  |  |
| --- | --- | --- | --- | --- | --- | --- | --- |
| PSEUDOWORD TARGETS | | | | | | | |
| wrups | jonde | guist | moufje | glantaart | switcht | drier | groest |
| opscho | fralong | kimps | zord | fikkelij | klepsje | smoere | herassen |
| jasser | sterrel | tuurs | rociem | gofo | soriek | banel | oplaf |
| nins | franiek | haide | schurs | stasoen | gantoor | noonts | aanpossel |
| twassie | kastroef | dwadel | troend | galfen | snirst | schrist | dwarseis |
| zastel | noetood | monoek | facent | foldong | treecht | swonts | wabond |
| skinder | doki | kokkung | hoosheid | ottart | joudee | tompel | spoonk |
| nehank | napster | staumi | snabel | ensem | juurs | ierreks | ziest |
| snastij | glores | celtaar | afonts | preciste | knierd | snart | heik |
| brindel | hepter | inpals | mokkon | fiest | kreel | nukker | saptje |
| dweesting | omest | zomps | hawel | stest | droorn | exalde | borenne |
| testop | taros | takes | gonak | schind | trodoon | nielam | gonide |
| klonku | stossen | sparla | sanduul | wollom | bahier | genading | goef |
| prars | bluniel | uitonds | pugeda | posast | lurd | plips | fruler |
| zwiel | flinkel | unks | plenar | klaaim | vonde | grienk | routie |
| gimps | woot | twistie | kapdor | tiffie | daffetje | dammo | ahoft |
| neman | fenim | strousef | dwompt | hontast | krouw | jekstje | binnel |
| wapt | vasok | erkst | sploort | lintsen | sliffer | pliekon | smeep |
| genaar | swinks | psong | spukisie | spruikje | spaam | mants | iniende |
| lanizie | vlasuur | plora | frood | plans | schompie | tronde | kundu |
|  |  |  |  |  |  |  |  |
