# Supplementary material for: When Feelings Arise with Meanings: How Emotion and Meaning of a Native Language Affect Second Language Processing in Adult Learners
Source: PLoS One. 2015 Dec 10;10(12):e0144576. doi: 10.1371/journal.pone.0144576 (PMC4684350; doi:10.1371/journal.pone.0144576)
Supplement: S1 Text — (DOCX) [file pone.0144576.s003.docx]

# S1 Text. Behavioral Results

This supplement presents additional analyses of the behavioral and EEG data for the German (L1) prime words and the Dutch (L2) targets: (1) Results of Sensitivity Rates; (2) Results of Accuracy Rates; (3) Results of Reaction Times. The alpha level adopted in all behavioral analyses is *p* < .05 (after Bonferroni correction for multiple comparisons).

**(1) Sensitivity Rates**

Word targets. A repeated measure ANOVA over sensitivity rates (*d’* scores) toward L2 word targets showed a significant main effect of session (*F* (3, 57) = 53.56, *p* < .001, η_p_^2^ = .74). Post-hoc comparisons confirmed that the sensitivity rate in Session 1 (mean = 2.03, SD = .53) was significantly lower than in Session 2 (mean = 2.9, SD = .7, *F* (1, 19) = 48.28, *p* < .001, η_p_^2^ = .72). The sensitivity rate in Session 2 was also significantly lower compared to Session 3 (mean = 3.16, SD = .62, *F* (1, 19) = 7.27, *p* = .014, η_p_^2^ = .28). However, the increase of sensitivity rate from Session 3 (mean = 3.16, SD = .62) to Session 4 (mean = 3.35, SD = .6, *F* (1,19) = 3.4, *p* = .08, η_p_^2^ = .15) was only marginally significant (see Figure 2).

Pseudoword targets. Another repeated measure ANOVA over sensitivity rates toward pseudoword targets also showed a significant main effect of session (*F* (3, 57) = 11.41, *p* < .001, η_p_^2^ = .38). A post-hoc comparison revealed a significant increase of sensitivity rate from Session 1 (mean = 1.61, SD = 1.07) to Session 2 (mean = 2.26, SD = 1.22, *F* (1, 19) = 7.24, *p* = .014, η_p_^2^ = .28). Another post-hoc comparison, however, showed that the increase of sensitivity rate from Session 2 to Session 3 (mean = 2.38, SD = 1.4, *F* (1, 19) < 1, *p* > .1, η_p_^2^ = .02) was not significant. As seen in Figure 2, the sensitivity rate significantly improved again from Session 3 to Session 4 (mean = 2.91, SD = 1.31, *F* (1, 19) = 7.13, *p* = .02, η_p_^2^ = .27).


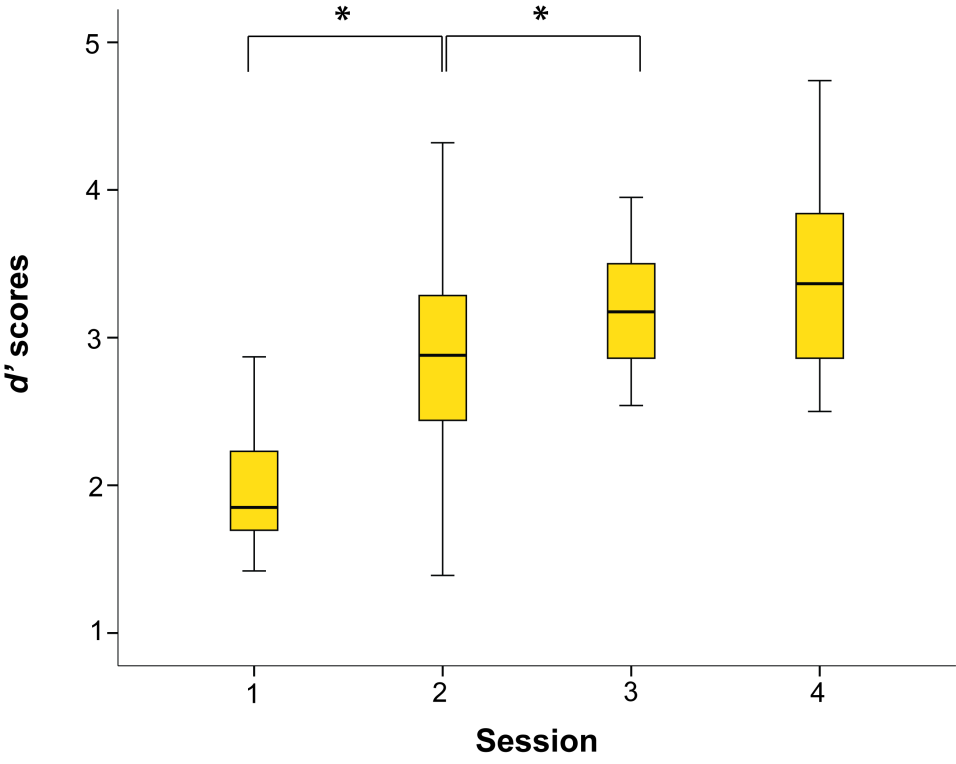


S1A Figure. The results of sensitivity rates (*d’* score) analysis over the four measurement sessions. Note: **p* < .05

## (2) L1-L2 priming effects on Accuracy Rates

##

A 4 (session) x 2 (semantic relatedness) x 2 (affective congruence) x 2 (target valence) repeated measures ANOVA over accuracy rates from 24 subjects revealed a significant main effect of semantic relatedness (*F* (1, 23) = 4.51, *p* = .045, η_p_^2^ = .16) indicating that more errors occurred in judging the lexicality of L2 targets following L1 unrelated primes (mean percentage = 94.17, SD = 2.83) than following L1 related primes (mean percentage = 94.74, SD = 2.48), which suggests that semantic associativeness between L1 primes and L2 targets plays an important role on the learner’s overall accuracy pattern. The ANOVA also showed a significant main effect of target valence (*F* (1, 23) = 25.4, *p* < .001, η_p_^2^ = .53) with a higher accuracy rate for L2 positive targets (mean percentage = 95.76, SD = 2.6) than L2 neutral targets (mean percentage = 93.15, SD = 3.1). In addition, there was a main effect of session (*F* (3, 69) = 48.53, *p* < .001, η_p_^2^ = .68) on the overall accuracy pattern, which was also corroborated by post-hoc pairwise comparisons between sessions. The learner’s accuracy rate in judging the lexicality of L2 targets was significantly higher in Session 2 (mean percentage = 95.78, SD = 2.85) than in Session 1 (mean percentage = 87.55, SD = 6.5, *F* (1, 23) = 38.88, *p* < .001, η_p_^2^ = .63). The accuracy rate for L2 word targets also increased in Session 3 (mean percentage = 97.32, SD = 1.82), which was significantly higher than the accuracy rate for L2 targets in Session 2 (*F* (1, 23) = 11.51, *p* = .003, η_p_^2^ = .33). However, the difference between the accuracy rates for L2 targets in Session 4 (mean percentage = 97.16, SD = .45) and Session 3 was not statistically significant (*F* (1, 23) < .1, *p* > .1, η_p_^2^ = .01). Finally, the interaction between affective congruence and target valence was only marginally significant (*F* (1, 23) = 3.3, *p* = .08, η_p_^2^ = .13). There were no significant main effect of affective congruency or other significant interaction effects on accuracy rates (all *p*s > .1).

## (3) L1-L2 priming effects on Reaction Times

Repeated measures ANOVAs revealed a significant main effect of semantic relatedness (*F* (1, 23) = 5.69, *p* = .03, η_p_^2^ = .2) showing somewhat shorter RTs for L2 targets following L1 related primes (mean = 588 ms, SD = 60 ms) than following L1 unrelated primes (mean = 593 ms, SD = 62 ms). The main effect of affective congruence (*F* (1, 23) = 6.76, *p* = .02, η_p_^2^ = .23) was also significant, reflecting that RTs for L2 targets following L1 congruent primes (mean = 588 ms, SD = 62 ms) were significantly shorter than following L1 incongruent primes (mean = 592 ms, SD = 60 ms). However, the interaction effects of semantic relatedness by affective congruence and of semantic relatedness or/and affective congruence by session were not significant (all *p*s > .1). Although we consider accuracy rate as the more important dependent variable in these learners, the RT results suggest that, across sessions, L1 primes influence response speed for L2 targets semantically and affectively in a constantly separate manner in the Early Learning Phase. The ANOVA also revealed a significant main effect of Session (*F* (3, 69) = 19.11, *p* < .001, η_p_^2^ = .45). Post-hoc comparisons showed that RT for L2 word targets in Session 2 (mean = 582 ms, SD = 73 ms) was significantly faster compared to those in Session 1 (mean = 652 ms, SD = 76 ms, *F* (1, 23) = 35.61, *p* < .001, η_p_^2^ = .61). RTs for L2 targets in Session 3 (mean = 575 ms, SD = 81 ms) was not significantly faster than those in Session 2 (*F* (1, 23) = .29, *p* > .1, η_p_^2^ = .01), and RT for L2 targets in Session 4 (mean = 552 ms, SD = 63 ms) was also not significantly faster compared to those in Session 3 (*F* (1, 23) = 1.89, *p* > .1, η_p_^2^ = .08). Finally, the ANOVA showed a significant 4-way interaction of Session, semantic relatedness, affective congruence, and target valence (*F* (3, 69) = 3.78, *p* = .01, η_p_^2^ = .14). Thus, the following post-hoc ANOVAs were carried out for every session.

Session 1. A post-hoc repeated measure 2 (semantic relatedness) x 2 (affective congruence) x 2 (target valence) ANOVA showed a significant main effect of affective congruence (*F*(1, 23) = 5, *p* = .035, η_p_^2^ = .18) with faster L2 targets following L1 congruent primes (mean = 650 ms, SD = 82 ms) than following L1 incongruent primes (mean = 654 ms, SD = 80 ms). The ANOVA also revealed a significant 3-way interaction of semantic relatedness, affective congruence, and target valence (*F* (1, 23) = 8, *p* = .01, η_p_^2^ = .26). A post-hoc comparison showed that L2 neutral targets (mean = 647 ms, SD = 79 ms) were responded faster than L2 positive targets (mean = 664 ms, SD = 80 ms) following L1 incongruent and unrelated primes. The RTs for the two types of L2 targets did not show any significant difference when preceded by other types of L1 primes (all *p*s > .1). The ANOVA showed no other main effects or significant interaction effects (all *p*s > .1).

Session 2. The post-hoc ANOVA showed only a tendency of an interaction between semantic relatedness and target valence (*F* (1, 23) = 3.4, *p* = .08, η_p_^2^ = .13). The ANOVA also showed no other main effects or significant interaction effects (all *p*s > .1).

Session 3. The post-hoc ANOVA showed a significant main effect of semantic relatedness reflecting that L2 targets following L1 related primes (mean = 571 ms, SD = 78 ms) were responded faster than those following L1 unrelated primes (mean = 579 ms, SD = 84 ms, *F* (1, 23) = 4.8, *p* = .04, η_p_^2^ = .17). There was also a significant interaction between semantic relatedness and target valence (*F* (1, 23) = 7.6, *p* = .01, η_p_^2^ = .25). Post-hoc comparisons showed a trend for L2 neutral targets (mean = 575 ms, SD = 87 ms) to be responded faster than L2 positive targets (mean = 582 ms, SD = 82 ms, *F* (1, 23) = 4.2, *p* = .05, η_p_^2^ = .15) following L1 unrelated primes. However, there was no difference between the RTs for L2 neutral and positive targets following L1 related primes (*p* > .1). The ANOVA showed no other main effects or significant interaction effects (all *p*s > .1).

Session 4. The post-hoc ANOVA only showed a marginally significant main effect of target valence (*F* (1, 23) = 3.4, *p* = .08, η_p_^2^ = .13). The ANOVA showed no other main effects or significant interaction effects (all *p*s > .1).

The 4-way interaction did not show any consistent pattern regarding Session, Semantic Relatedness, Affective Congruence, and Target Valence effects. Hence, we would not discuss these results; rather we want to focus on the ERP analysis as the more sensitive measure on the time-course of word processing.

## 
