# Supplementary material for: When Feelings Arise with Meanings: How Emotion and Meaning of a Native Language Affect Second Language Processing in Adult Learners
Source: PLoS One. 2015 Dec 10;10(12):e0144576. doi: 10.1371/journal.pone.0144576 (PMC4684350; doi:10.1371/journal.pone.0144576)
Supplement: S2 Text — (DOCX) [file pone.0144576.s004.docx]

# S2 Text. ERP analyses of the primes and word-pseudoword targets

This supplement presents additional analyses of the EEG data for the German (L1) prime words and the Dutch (L2) targets: (1) Analysis of the LPC component of the primes; (2) Analysis of the N400 component of word and pseudoword targets.

## (1) Analysis of LPC (430-550 ms) for the primes

A repeated measure 4 (Session) x 6 (Quadrant) x 3 (Valence) ANOVA showed a significant interaction of Quadrant and Valence (*F* (10, 190) = 9.1, *p* < .001, η_p_^2^ = .32). Follow-up analyses revealed a tendency of valence effect on LPC amplitudes over the left central quadrant (*F* (2, 38) = 2.8, *p* = .07, η_p_^2^ = .13). The valence effect was significant at the left posterior (*F* (2, 38) = 7.48, *p* < .01, η_p_^2^ = .28) and right posterior quadrant (*F* (2, 38) = 8.1, *p* < .01, η_p_^2^ = .3). More post-hoc comparisons over amplitudes at the left posterior showed that the LPC amplitudes elicited by neutral primes (mean = -.62 μV, SD = 1.8 μV) did not significantly differ from those elicited by negative primes (mean = -.8 μV, SD =1.9 μV, *F* (1, 19) = 3.2, *p* > .05, η_p_^2^ = .15), but it was significantly reduced than those elicited by positive primes (mean = -.36 μV, SD = 2 μV, *F* (1, 19) = 4.6, *p* < .05, η_p_^2^ = .19). The amplitude difference between negative primes and positive primes were also significant (*F* (1, 19) = 13.4, *p* < .01, η_p_^2^ = .41). At the right posterior quadrant, neutral primes (mean = -1.2 μV, SD = 1.7 μV) elicited larger LPC than did negative primes (mean = -1.5 μV, SD = 1.7 μV, *F* (1, 19) = 11.8, *p* < .01, η_p_^2^ = .38), but similar to the LPC elicited by positive primes (mean = -1.1 μV, SD = 1.9 μV, *F* (1, 19) < .1, *p* > .1). Finally, a post-hoc comparison showed that positive primes elicited larger LPC amplitudes than did negative primes (*F* (1, 19) = 12.6, *p* < .01, η_p_^2^ = .4). A separate 4 (Session) x 3 (Valence) over ERP amplitudes at the midline site did not show any significant main effect of Target Valence (*F* (2, 38) < .1, *p >* .1). The ANOVAs did not show any other significant main effects or interactions (all *p*s > .1).

Hence, results from the ERPs of the primes showed that there was an LPC effect driven by Prime’s valence at the posterior sites. Specifically, positive L1 words showed outstandingly larger positivity compared to neutral (at the left hemisphere) and negative ones (at both hemispheres), which is in line with previous ERP findings in single-word processing studies. The results clearly suggested that our participants attended to the emotional content of the primes.

## (2) Analysis of N400 (350-550 ms) for word and pseudoword targets

A repeated measure 4 (session) x 6 (quadrant) x 2 (target type) showed a significant Session effect (*F* (3, 57) = 6.8, *p* =.001, η_p_^2^ = .26). There were also a significant main effect of quadrant (*F* (5, 95) = 18.26, *p* < .001, η_p_^2^ = .49), target type (*F* (1, 19) = 188.35, *p* < .001, η_p_^2^ = .91), and a significant interaction of quadrant and target type (*F* (5, 95) = 11.5, *p* < .001, η_p_^2^ = .38). Post-hoc comparisons showed reduced N400 amplitude for word targets (mean = .33 μV, SD = .21 μV) relative to the N400 amplitude for pseudoword targets (mean = .13 μV, SD = .21 μV) across sessions. More post hoc comparisons confirmed that the N400 effect on lexicality was significant at the left (mean word = 1.13 μV, SD = 2.4 μV, mean pseudoword = -1.14 μV, SD = 2.5 μV, *F* (1, 19) = 93.4, *p* <.001, η_p_^2^ = .83) and right anterior (mean word = 1.9 μV, SD = 2.5 μV, mean pseudoword = -1.4 μV, SD = 2.3 μV, *F* (1, 19) = 171.83, *p* <.001, η_p_^2^ = .90). There were also a significant N400 effect on targets’ ERPs at the left central (mean word = 3.7 μV, SD = 3.3μV, mean pseudoword = -.7 μV, SD = 3 μV, *F* (1, 19) = 118, *p* < .001, η_p_^2^ = .86), right central (mean word = 3.5 μV, SD = 3.1 μV, mean pseudoword = -.7 μV, SD = 2.7 μV, *F* (1, 19) = 199.5, *p* < .001, η_p_^2^ = .91), left posterior (mean word = 4.7 μV, SD = 3.5 μV, mean pseudoword = 1.1 μV, SD = 3 μV, *F* (1, 19) = 101.8, *p* < .001, η_p_^2^ = .84), and right posterior quadrants (mean word = 4.6 μV, SD = 3.4 μV, mean pseudoword = 1.3 μV, SD = 2.9 μV, *F* (1, 19) = 191.96, *p* < .001, η_p_^2^ = .91). Finally, the interaction between session and target type was significant (*F* (3, 37) = 4.3, *p* < .01, η_p_^2^ = .18). Post hoc comparisons showed that the N400 effects were significant at Session 1 (mean word = 2 μV, SD = 2.6 μV, mean pseudoword = -.9 μV, SD = 2.4 μV, *F* (1, 19) = 111.6, *p* < .001, η_p_^2^ = .86), Session 2 (mean word = 3.4 μV, SD = 3.5 μV, mean pseudoword = -.4 μV, SD = 2.7 μV, *F* (1, 19) = 147.8, *p* < .001, η_p_^2^ = .88), Session 3 (mean word = 3.9 μV, SD = 3.1 μV, mean pseudoword = .02 μV, SD = 2.6 μV, *F* (1, 19) = 141, *p* < .001, η_p_^2^ = .88), and Session 4 (mean word = 3.7 μV, SD = 2.7 μV, mean pseudoword = .04 μV, SD = 2.7 μV, *F* (1, 19) = 116.5, *p* < .001, η_p_^2^ = .86). At the midline site, a separate 4 (session) x 2 (target type) ANOVA showed a significant main effect of session (*F* (3, 57) = 5.28, *p* < .01, η_p_^2^ = .22), target type (*F* (1, 19) = 188.75, *p* < .001, η_p_^2^ = .91), wherein pseudoword targets (mean = .01 μV, SD = .87 μV) elicited larger N400 than did word targets (mean = 1.1 μV, SD = .85 μV). The interaction between session and target type was also significant (*F* (3, 57) = 2.84, *p* < .05, η_p_^2^ = .13). Post hoc comparisons showed that the N400 effects were significant at Session 1 (mean word = 2.4 μV, SD = 2.9 μV, mean pseudoword = -1.7 μV, SD = 3 μV, *F* (1, 19) = 135, *p* < .001, η_p_^2^ = .88), Session 2 (mean word = 4.2 μV, SD = 4.4 μV, mean pseudoword = -1 μV, SD = 3.7 μV, *F* (1, 19) = 153.4, *p* < .001, η_p_^2^ = .89), Session 3 (mean word = 4.4 μV, SD = 3.8 μV, mean pseudoword = -.6 μV, SD = 3.4 μV, *F* (1, 19) = 148.7, *p* < .001, η_p_^2^ = .88), and Session 4 (mean word = 4.1 μV, SD = 3.8 μV, mean pseudoword = -.55 μV, SD = 3.6 μV, *F* (1, 19) = 116.5, *p* < .001, η_p_^2^ = .86).
